# Dengue in Myanmar: Spatiotemporal epidemiology, association with climate and short-term prediction

**DOI:** 10.1371/journal.pntd.0011331

**Published:** 2023-06-05

**Authors:** Win Zaw, Zaw Lin, July Ko Ko, Chawarat Rotejanaprasert, Neriza Pantanilla, Steeve Ebener, Richard James Maude

**Affiliations:** 1 Mahidol Oxford Tropical Medicine Research Unit, Faculty of Tropical Medicine, Mahidol University, Bangkok, Thailand; 2 Vector Borne Disease Control, Department of Public Health, Ministry of Health, Nay Pyi Taw, Myanmar; 3 Department of Tropical Hygiene, Faculty of Tropical Medicine, Mahidol University, Bangkok, Thailand; 4 Centre for Tropical Medicine and Global Health, Nuffield Department of Medicine, University of Oxford, Oxford, United Kingdom; 5 Harvard TH Chan School of Public Health, Harvard University, Boston, Massachusetts, United States of America; 6 The Open University, Milton Keynes, United Kingdom; University of California Berkeley School of Public Health, UNITED STATES

## Abstract

Dengue is a major public health problem in Myanmar. The country aims to reduce morbidity by 50% and mortality by 90% by 2025 based on 2015 data. To support efforts to reach these goals it is important to have a detailed picture of the epidemiology of dengue, its relationship to meteorological factors and ideally to predict ahead of time numbers of cases to plan resource allocations and control efforts. Health facility-level data on numbers of dengue cases from 2012 to 2017 were obtained from the Vector Borne Disease Control Unit, Department of Public Health, Myanmar. A detailed analysis of routine dengue and dengue hemorrhagic fever (DHF) incidence was conducted to examine the spatial and temporal epidemiology. Incidence was compared to climate data over the same period. Dengue was found to be widespread across the country with an increase in spatial extent over time. The temporal pattern of dengue cases and fatalities was episodic with annual outbreaks and no clear longitudinal trend. There were 127,912 reported cases and 632 deaths from 2012 and 2017 with peaks in 2013, 2015 and 2017. The case fatality rate was around 0.5% throughout. The peak season of dengue cases was from May to August in the wet season but in 2014 peak dengue season continued until November. The strength of correlation of dengue incidence with different climate factors (total rainfall, maximum, mean and minimum temperature and absolute humidity) varied between different States and Regions. Monthly incidence was forecasted 1 month ahead using the Auto Regressive Integrated Moving Average (ARIMA) method at country and subnational levels. With further development and validation, this may be a simple way to quickly generate short-term predictions at subnational scales with sufficient certainty to use for intervention planning.

## Author summary

The study describes country, State/Region and township level spatial and temporal epidemiology of dengue in Myanmar from routine surveillance data collected by the government. Dengue was found to widespread across the country with an increase in spatial extent over time, likely due to improvements in surveillance, reporting and access to health services. Most reported cases were children with an increase in the proportion ≥15 years of age over time. Dengue incidence was seasonal and related to climate, with larger annual peaks every two years, although the timing of peaks in incidence, strength and nature of the relationship with climate differed between States and Regions. Using ARIMA, it was possible to predict dengue incidence 1 month ahead at State/Region level using previous incidence data. Adding monthly climate data to the model gave a marginal improvement in model performance which did not justify the additional effort needed to acquire and process it. With further development and validation, the method may be a simple way to quickly generate short-term predictions at subnational scales with sufficient certainty to use for intervention planning.

## Introduction

Dengue is a tropical and sub-tropical mosquito-borne arboviral disease. The World Health Organization (WHO) has estimated that 100–400 million people are infected with dengue each year [1] and half of the world population are exposed to it. There has been a steady increasing trend of dengue incidence globally since at least 1990 [2], with around 70% of the reported burden [1] and 75% of the population exposed being in the Asia-Pacific region.

Dengue is transmitted from human to human by *Aedes* mosquito bites and humans are the main host. *Ae*. *aegypti* is the most efficient vector species and the female mosquito bites humans during the day. The infected female can transmit dengue immediately by biting another host when its feeding is interrupted [3] or after an incubation period of 8–10 days during which time the virus multiplies in the salivary glands. The infected mosquito host remains active for 30–45 days [4]. Many factors affect the transmission of dengue, such as entomological, socio-economical, environmental and demographic [5]. Most of these are difficult to measure, not available on a continuous basis and thus of limited use in real-time predictions to inform dengue control activities. Human movement has been shown to help predict dengue incidence as it causes virus spread across diverse geographical scales [6–9] despite limited dispersal ranges of *Aedes* mosquitoes [10, 11]. However, such data are not generally available for routine use and modelling methods can be complex. The relationship between dengue incidence and climate factors has been well established with rainfall, higher temperatures and humidity being associated with increased mosquito numbers and dengue transmission. [12] Climate data is collected routinely at weather stations as well as through satellite remote sensing and could potentially be used to develop real-time dengue prediction models. However, the relationship between climate and dengue incidence is not consistent across time and space [13–16] and location-specific models are generally needed.

Myanmar is hyperendemic for dengue with all four known dengue serotypes and it is among the top ten childhood diseases for hospitalization in the country [17]. Dengue was first reported in Yangon in 1964 with the first major outbreak occurring in 1970 and spreading to all other States and Regions. Yangon Region continues to have the highest incidence reported in the country. Over time, dengue cases have spread to more townships and outbreak frequency has increased [18]. Dengue cases are reported routinely by hospitals across the country to the Vector Borne Disease Control Programme (VBDC) but there is limited capacity for fine-scale analysis of these data to inform planning of dengue control activities. The country aims to reduce dengue morbidity by 50% and mortality by 90% by 2025 based on 2015 data [18] and making better use of existing data may help move towards this goal. As found elsewhere, one or more of temperature, rainfall and humidity may be associated with dengue incidence, with a time lag for mosquito breeding and disease transmission to occur, thus giving the possibility to predict dengue incidence from climate data.

This study aimed to better understand the patterns of dengue over space and time in Myanmar and their relationship to routinely collected climate data. This was to provide evidence to assess the utility of these data for a weather-based early warning system for dengue.

## Methods

### Ethics statement

This study used anonymized, aggregated secondary data thus formal ethical approval was not required and a waiver was provided by the Oxford Tropical Research Ethics Committee (OxTREC) and no additional ethical approval was required by the Myanmar government. Case data were obtained with permission from the Dengue Control Unit of the Ministry of Health and Sports, Myanmar.

### Data

Monthly health facility-based dengue incidence data aggregated at township level were collected from the National Dengue Control Programme, VBDC, Ministry of Health and Sports from 2012 to 2017. Data had already been cleaned by VBDC staff. This comprised numbers of reported hospitalized dengue haemorrhagic fever cases (DHF), graded from I to IV; grades III and IV being classed as dengue shock syndrome (DSS). State and Region level data were available by month and township level data were available by year. Diagnostic criteria for dengue in Myanmar followed the 1997 World Health Organization guidelines [19]. Analysis by age group was limited to the age bands in the surveillance database and mortality by age was only available for 2016–2017. Dengue surveillance in Myanmar is primarily hospital-based, thus community cases are not captured. Population data were the total population counts for each area from the most recent census done in 2014.

### Weather data

It was not possible to access monthly climate station data directly from the government. Therefore climate station based daily minimum temperature, maximum temperature, mean temperature and DEW point temperature, available from Jan 2012 to Dec 2017 for Yangon and to May 2016 for other areas, were downloaded from the NOAA National Climatic Center, US Government [20]. Rainfall was not available for Myanmar for this time period from NOAA. Rasterized rainfall layers were available at 0.5 by 0.5 degree (approx. 50 by 50 km) resolution for Jan 2012 to Dec 2017 and were downloaded from the Climatic Research Unit, (CRU) University of East Anglia website [21]. From the CRU, rasterized temperature was also available, but only monthly average values. Percent humidity was calculated from DEW point temperature and mean temperature [22]. From the climate station dataset, thirty-two ground-based weather stations in Yangon, Mandalay, Ayeyarwady, Kachin, Kayah, Rakhine, Sagaing and Tanintharyi Regions were used for analysis and correlation with monthly dengue incidence. Where multiple stations were present in an administrative unit, values were averaged. Due to missing station data, Bago, Magway, Mon, Kayin and Shan States were omitted from this analysis. Both ground-based weather data and rasterized rainfall data were aggregated at monthly level to match the dengue incidence data.

### Statistical analysis

The data were cleaned and geo-coded at the State and Region and Township levels. Spearman ρ correlation analysis and graphs of State/Region level morbidity and mortality were produced using GraphPad Prism version 8.0.0 for Windows, GraphPad Software, La Jolla California USA. Township level population risk maps and case fatality maps were produced using ArcGIS version 10.3 (ESRI, CA). Raster data were aggregated as monthly average values for the cells falling within the relevant administrative unit polygons.

Seasonality and trend of the log transformed dengue incidence were examined using the multiplicative time series decomposition function in the *forecast* package in R Studio (1.1.383). [23] This splits the data series into seasonality, trends and random fluctuation. The equation can be written as follows

$$logY_{t}=logS_{t}\times logT_{t}\times logR_{t}$$

Where *Yt* is dengue incidence, *St* is the seasonal component, *Tt* is the trend cycle of the disease and *Rt* is the remainder component.

Monthly dengue case counts and monthly weather variables from each State/Region from 2012 to 2017 were used for time series analysis using distributed lag non-linear models (DLNM). To capture short-term non-linear associations, a univariate model with quasi-Poisson family and log link was used to monitor dengue case counts. The DLNM was first applied to each State/Region, tested for dengue incidence and their association with each of (mean, minimum and maximum) temperature, total rainfall and relative humidity. The DLNM terms have two dimensions, the exposure space and the lag space that allows estimation of non-linear effect of climate variables at each lag and non-linear effect across all lags [24]. The DLNM was perform using the *dlnm* package in R Studio (1.1.383). To look for the non-linear effect of climate variables, we used “natural cubic spline” using a 3*df* and the lagged effect using a 3*df* natural cubic spline. A maximum lag of 10 months was used to ensure inclusion of a plausible range of lag periods inferred from previous studies showing dengue transmission is highly sensitive to climate [15, 25, 26].

In the second stage, to account for confounding, different combinations of meteorological variables were modelled together in a multivariable analysis using the same two DLNM terms. Each climate variable was tried in turn as the exposure variable with adjustment for the other climate variables. The best model for each State/Region was then selected based on QAIC (Quasi-Akaike information criteria) as the quasi-likelihood is assumed for distributed lag modeling. In this stage, due to multi-collinearity [27] issues and some states having a high proportion of zero values (zero inflation), it was only possible to produce results for a some States/Regions and using only limited subsets of variable combinations.

### Dengue incidence forecasting

Autoregressive integrated moving average (ARIMA) [28] modelling was used to predict monthly dengue incidence at country and at State/Region levels. The ARIMA model uses historic incidence data to predict future trends, is relatively simple to use for programmatic purposes like dengue incidence prediction [29–31] and has been shown to have more predictive power than other models [32, 33]. ARIMA models were written in *ARIMA (p*, *d*, *q)* where p is the order of the auto-regressive (*AR*) mode is a regression model with values of y until P^th^ time as predictors also known as lag order and **ε** is the white noise at time ***t*** and ***c*** is a constant and **φ** are parameters.

d is the degree of differencing: the number of times raw observations are differenced until the original data becomes stationery and q is the order of moving average (*MA*) of the model on past forecast errors where ε is white noise at time t and c is the constant, θs are parameters. The combination of all three models resulted in the ARIMA(p,d,q) model, as below:

$${\hat{y}}_{t}^{\prime }=c+\emptyset_{1}y_{t-1}^{\prime }+\emptyset_{2}y_{t-2}^{\prime }+\cdots +\emptyset_{p}y_{t-p}^{\prime }+\theta_{1}\epsilon_{t-1}+\theta_{2}\epsilon_{t-2}+\cdots +\theta_{q}\epsilon_{t-q}+\epsilon_{t}$$

The model used the *auto*.*arima ()* method [23] in the *forecast* package in R Studio (1.1.383) which chooses the best ARIMA model order [23]. The function in R uses a variation of the Hyndman-Khandakar algorithm and automatically selects the best ARIMA order. [28] This was used as we compared a large number of models with different parameter specifications and temporal lags. An algorithm which can be operated automatically was felt to be more appropriate for our purpose. Studies of automatic algorithms showed this methodology to be particularly good for short-term prediction. [28, 34] In addition, this method is implemented in a free software package (R language) which is cost efficient and could potentially be further utilized by the government for their dengue surveillance data analysis. The proposed algorithm for the automatic ARIMA model is where the number of differences ***0≤d≤2*** is determined using repeated KPSS (Kwiatkowski–Phillips–Schmidt–Shin) tests, and the values of ***p*** and ***q*** are selected by minimizing the **AIC** (Akaike information criteria) after differencing the data ***d*** times. Using this method, short-term predictions of 1 month ahead for national and sub-national levels that included three States/Regions (Yangon, Ayeyarwady, Mandalay) with the highest dengue incidence were analyzed using data from preceding months. This 1 month ahead timescale was selected in consultation with the national dengue control programme after preliminary analysis showed 2–3 month ahead predictions to be much less accurate. The variable used for forecasting was the monthly dengue incidence per 100,000 population which was log transformed to stabilize variance. Models were run with and without all possible combinations of weather variables with lags from 0 to 3 months.

The data were plotted and examined for unusual observations. The residuals of the model were checked for white noise with an ACF (Auto Correlation Function) plot. If it did not look like white noise, the ACF plot of the differenced data was checked. If the residuals were white noise, the models were processed for forecasting.

The data were split into a training period from the start of the data set to 12 months before the end and testing period of the final 12 months. Forecast accuracies were evaluated by calculating mean squared errors (MSE), which is the difference between observed data and predicted data in the testing section and predictive R^2^, which is the variance of time series. The residuals graphs and ACF plot were also used to check the accuracy of the models [23]. Forecasts were plotted with 95% confidence Intervals (CI) and visually compared with observed data.

## Results

### Country level

**Fig 1:**
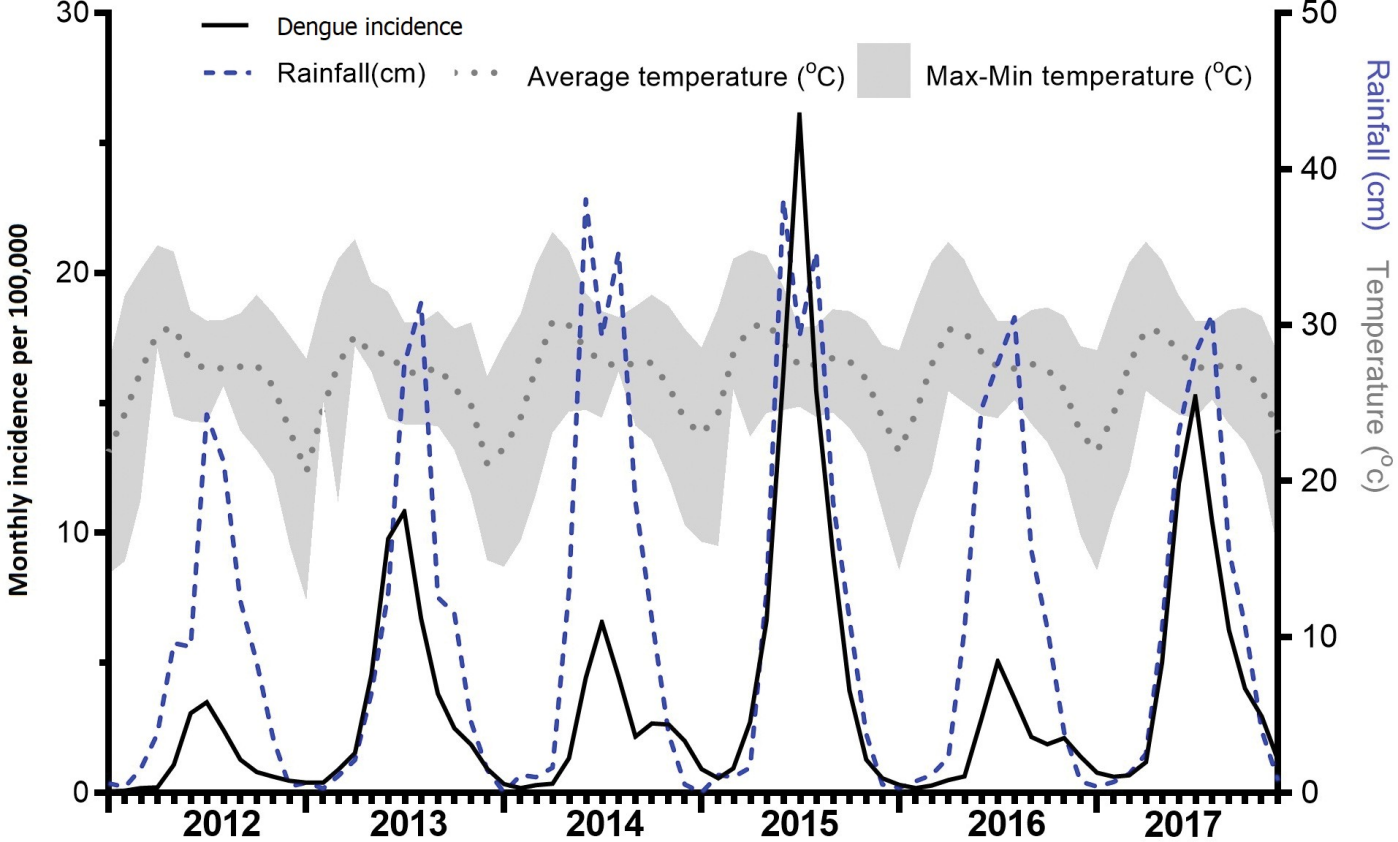
Seasonal pattern of dengue, rainfall and temperature.

**Fig 2:**
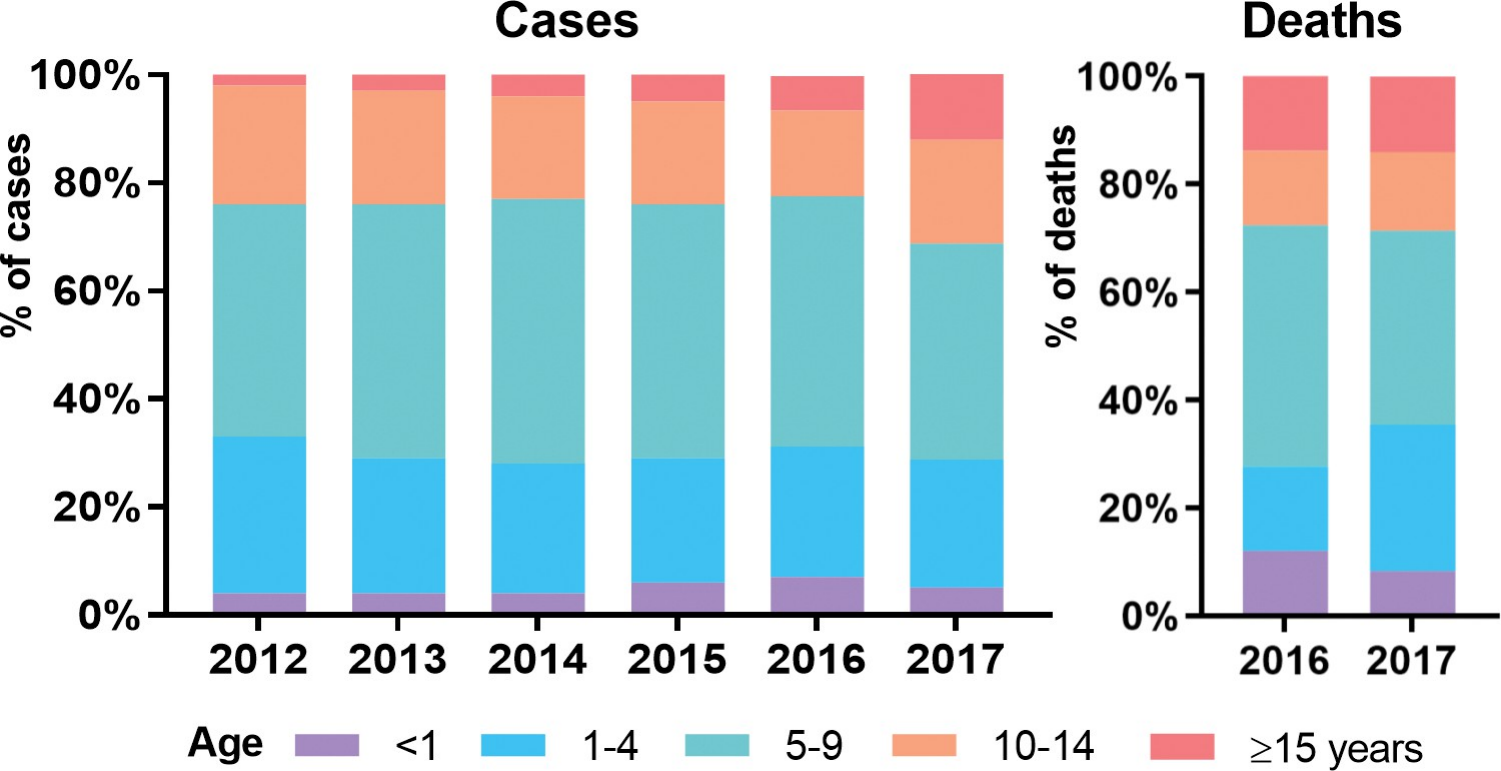
Proportion of dengue cases from 2012 to 2017 and deaths in 2016 and 2017 by age group.

From 2012 to 2017, 127,912 dengue cases were reported. During this period, there were 632 deaths with annual case fatality rates (CFR) of 0.45%, 0.54%, 0.65%, 0.33%, 0.54% and 0.61%, an average of 0.50%. There was no trend over time in reported cases or CFR but there were peaks in incidence in 2013, 2015 and 2017 (‘peak years’, **Fig 1**). Eighty nine percent of suspected cases and 86% of deaths were aged 15 years or less (5.6% <1 year, 24% 1–4 years, 42% aged 5–9 years, 18% 10–14 years and 11%>15 years; deaths 6% <1, 24% 1–4, 38% 5–9, 14% 10–14 and 14% >15 years, **Fig 2**). The 5–9 year age group contributed the highest proportions of both incidence (42% overall) and mortality (38%). 49.8% of suspected cases were male with CFRs 0.4% in males and 0.7% in females in 2016 and 2017 (the only years for which deaths were available by gender). There was a gradual increase in the proportion 15 years or older from 2.0% in 2012 to 12.0% in 2017 but there were no differences in age or gender breakdown between peak and non-peak years. Historically, dengue was mostly found in urban areas in Myanmar. In the data, the disease was equally present in both rural and urban areas (**Fig A in S1 Text**) with an increase in the proportion of urban cases in 2017.

### State and region level

**Fig 3:**
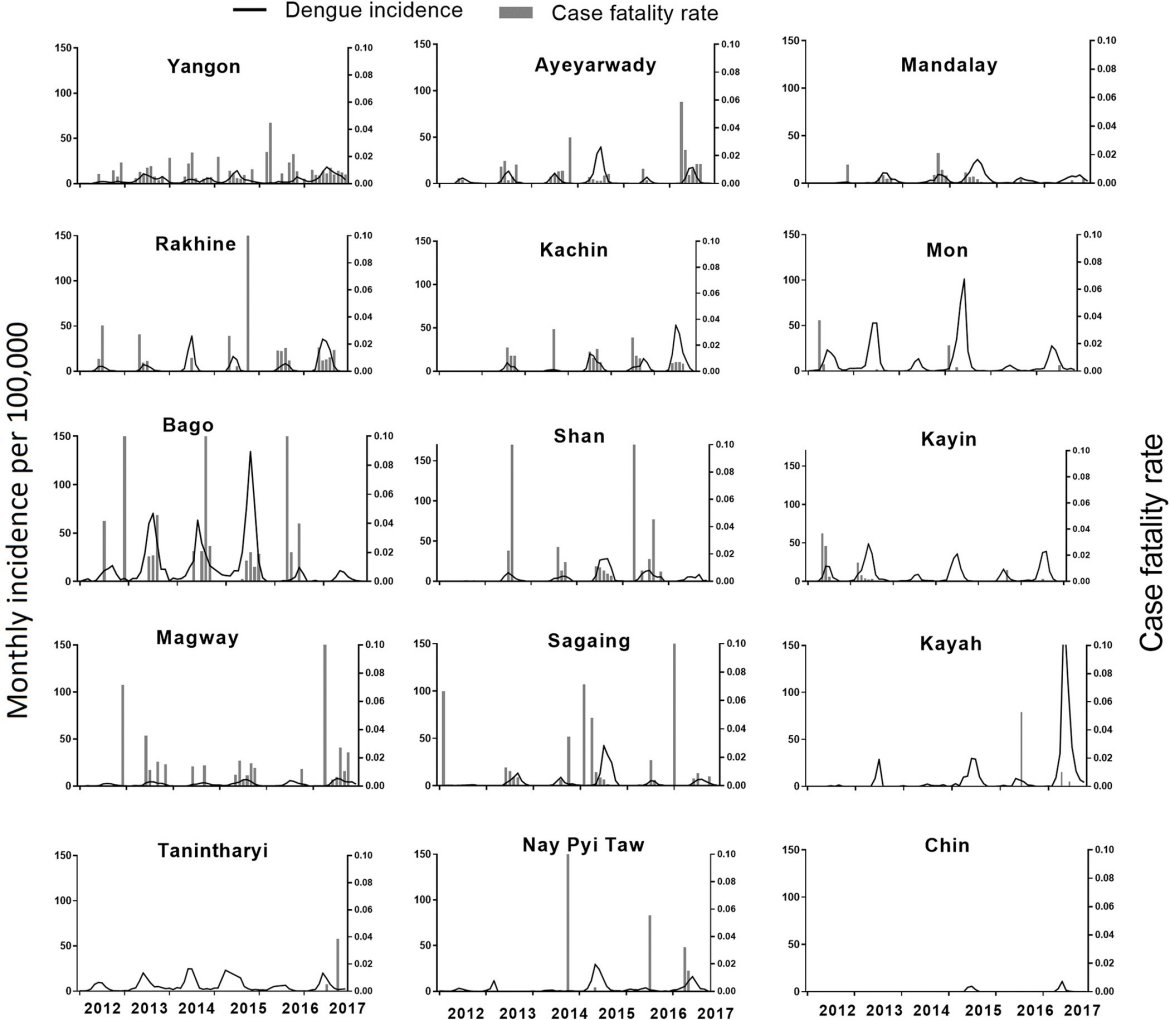
Monthly dengue incidence and mortality for States and Regions from 2012 to 2017. Left Y axis: Incidence per 100,000 population in descending order of overall case count. Right Y axis: Case fatality rate.

Dengue was reported in all fourteen States and Regions and Nay Pyi Taw council area. There was a strong seasonal pattern of dengue cases and deaths, both with peaks in July to September (**Figs 3 and B in S1 Text**). The timing of peaks in incidence varied greatly between States and Regions with the same seasonal pattern but peak years/outbreaks in different years in different locations (**Fig 3**). Mon State had the highest average incidence rate, followed by Kayah, Kayin and Tanintharyi (**Table A in S1 Text**). The highest percentages of total cases were in Yangon with 17%, Ayeyarwaddy with 12%, Mandalay with 11% and Mon with 11%. In 2015, cases were reported for the first time in Chin State (**Table A in S1 Text**). In 2015, Kayah State recorded the highest incidence rate (384 cases per 100,000 population) during the study period. The highest number of deaths was reported in Yangon Region (60 deaths in 2017; 31.3% of the annual total with CFR 0.8%). The highest CFR was in Shan North Region at 2.4% in 2013 (**Table B in S1 Text**). Severity of reported dengue cases varied by State and Region with most cases in Mandalay Region being Grade II but all other States and Regions being mostly Grade I (**Fig C in S1 Text**). The proportion with DSS varied greatly across the country. Severity data were only available from 2016 and 2017 but there was consistency in these patterns in these two years.

### Township level

**Fig 4:**
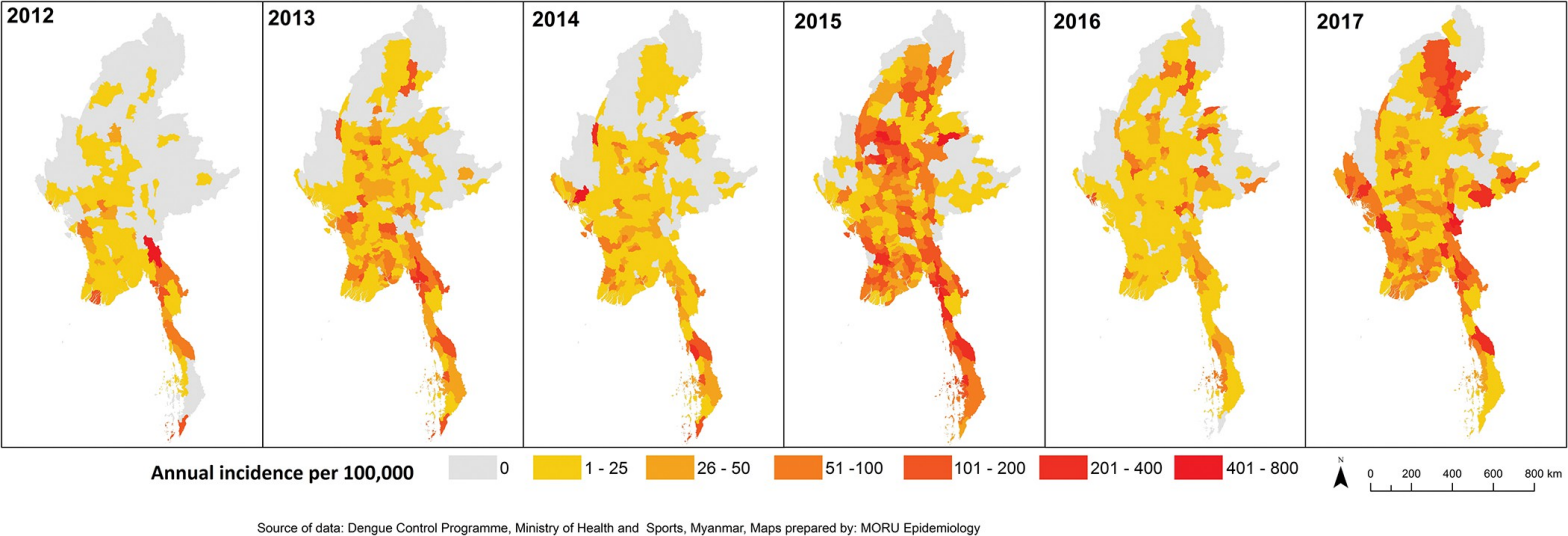
Township level annual dengue incidence per 100,000 population from 2012 to 2017. Source and terms of use for shapefile: https://geonode.themimu.info/layers/geonode%3Ammr_polbnda_adm3_250k_mimu.

**Fig 5:**
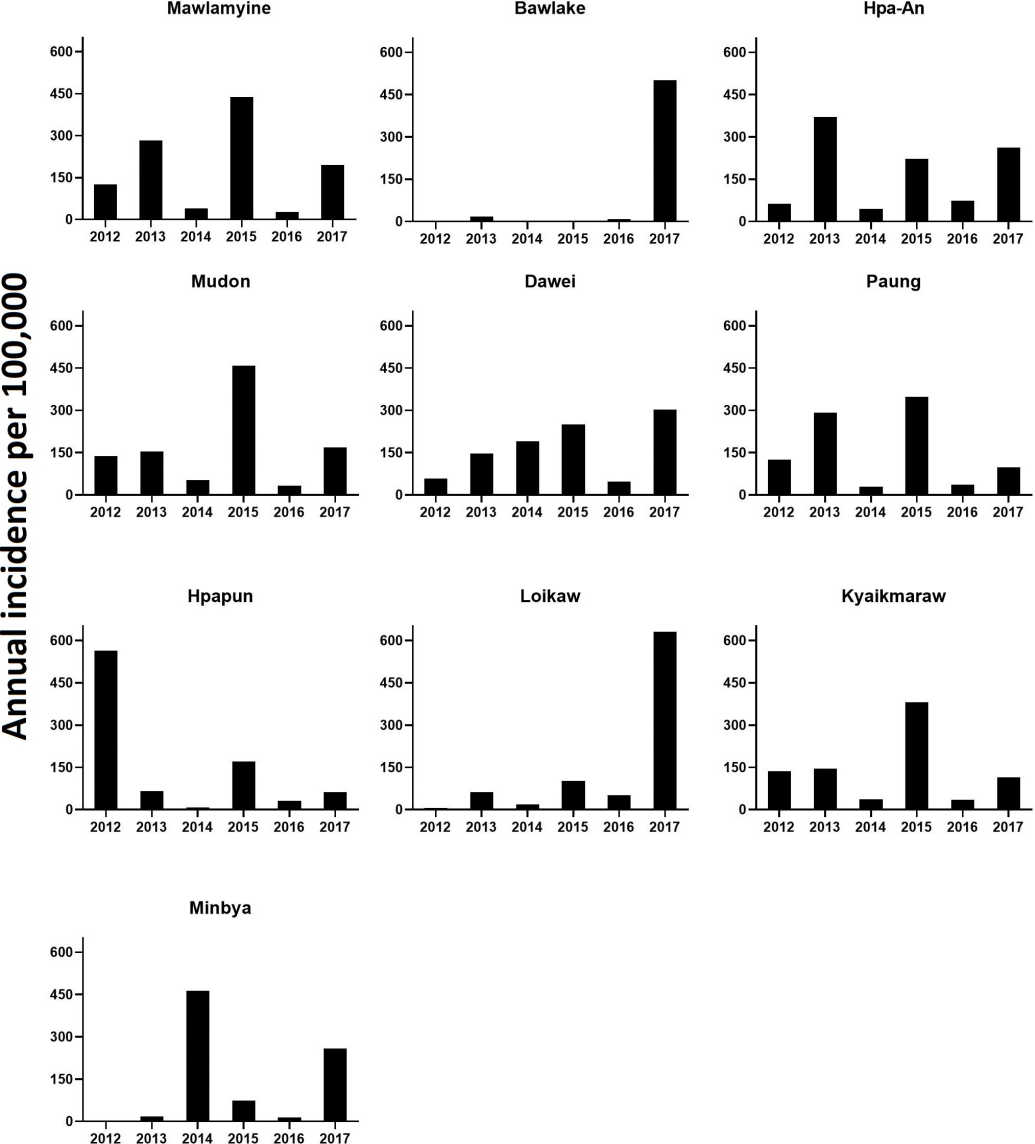
Top ten endemic townships with annual dengue incidence per 100,000 population from 2012 to 2017.

In 2012, 195/330 townships reported dengue cases, increasing to 252, 253, 272, 304 and 292 from 2013 to 2017. (**Fig 4**). There was significant annual spatial variation in the distribution of dengue cases with an overall spread into northern, eastern and western townships over time. The townships with highest incidence varied from year to year with peaks in different years in different townships and no clear pattern of maximum incidence across townships (**Fig 5**). In 2017, townships in the northern and eastern parts of the country had the highest incidence overall (**Fig 4**).

The 5 townships with the highest annual average incidence rates were Mawlamyine (185/100,000) in Mon State, Bawlake (176/100,000) in Kayah State, Hpa-An (172/100,000) in Kayin State, Dawei in Tanintharyi Region (166/100,000) and Minbya in Rakhine State (138/100,000) (**Fig 5**). The next five highest incidence rates were two townships in Mon State and one township each from Kayin, Kayah and Rakhine State with more than 100 cases per 100,000.

### Case fatality rate (CFR)

**Fig 6:**
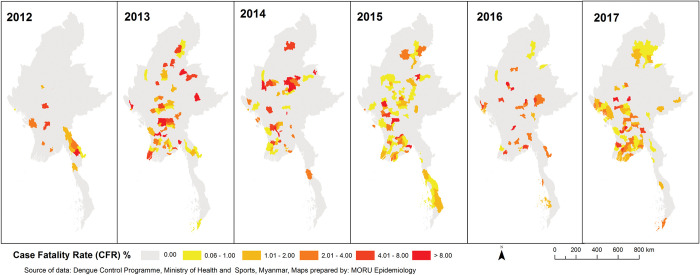
Township level annual dengue case fatality rates from 2012–2017. Source and terms of use for shapefile: https://geonode.themimu.info/layers/geonode%3Ammr_polbnda_adm3_250k_mimu.

CFRs varied widely between townships with a varying geographical distribution from year to year (**Fig 6**). The number of townships reporting fatal cases increased from 19 in 2012 to 99 in 2017, with average 72 (26% of townships with cases) from 2012 to 2017. There was no clear pattern in the spatial distribution of CFRs at township level, however increased incidence generally coincided with increases in numbers of deaths.

### Seasonal Pattern of Dengue incidence

**Fig 7:**
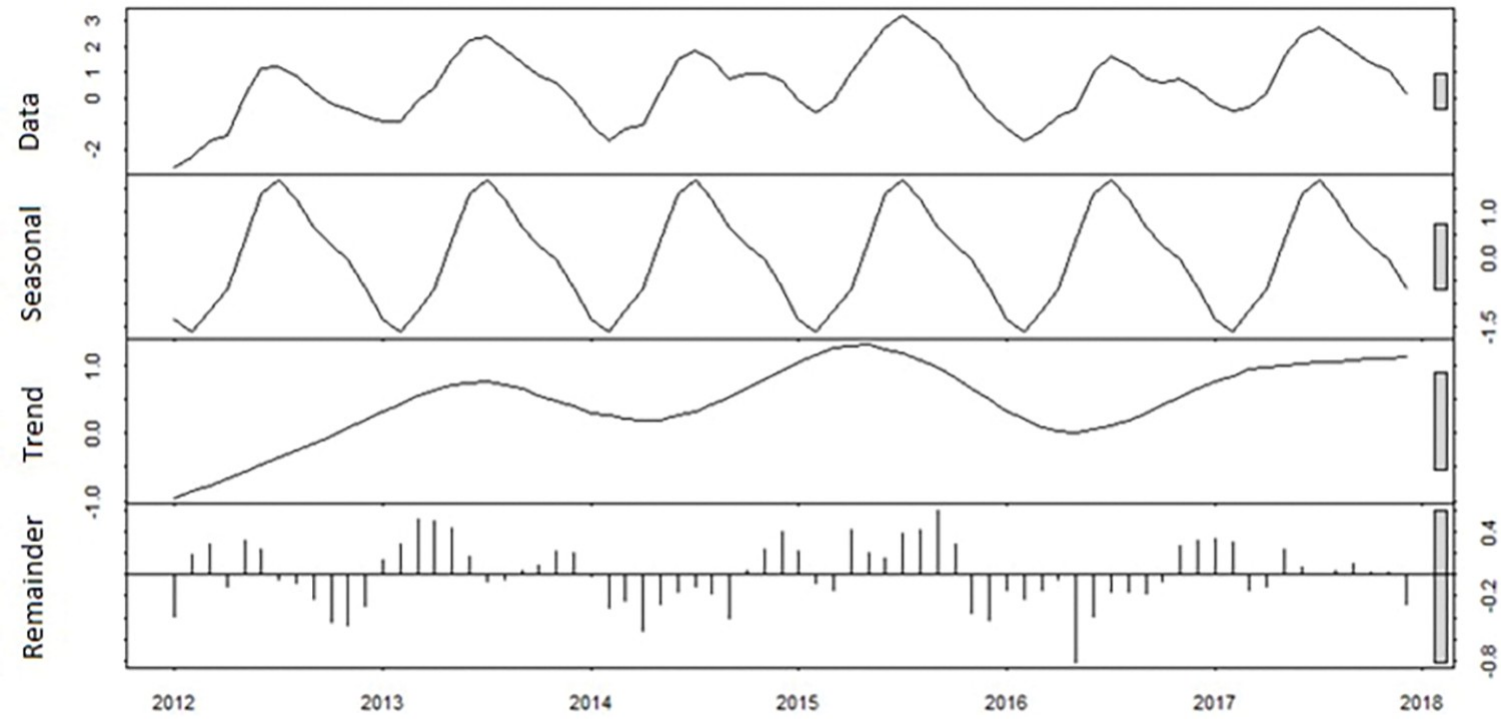
Decomposition of additive time series of dengue cases from 2012–2017. From top to bottom: reported incidence, trend, seasonality and random component.

A seasonal pattern was observed with annual peak from June to September and highest incidence in July. This followed the peak in temperature by 2 to 3 months and coincided with, or preceded, the peak in rainfall by 1–2 months. The rainy season is from May to October each year. Time series decomposition showed there was strong seasonal pattern each year with no trend over time, a peak every 2 years and no changes in seasonality (**Fig 7**).

### Serotypes

**Fig 8:**
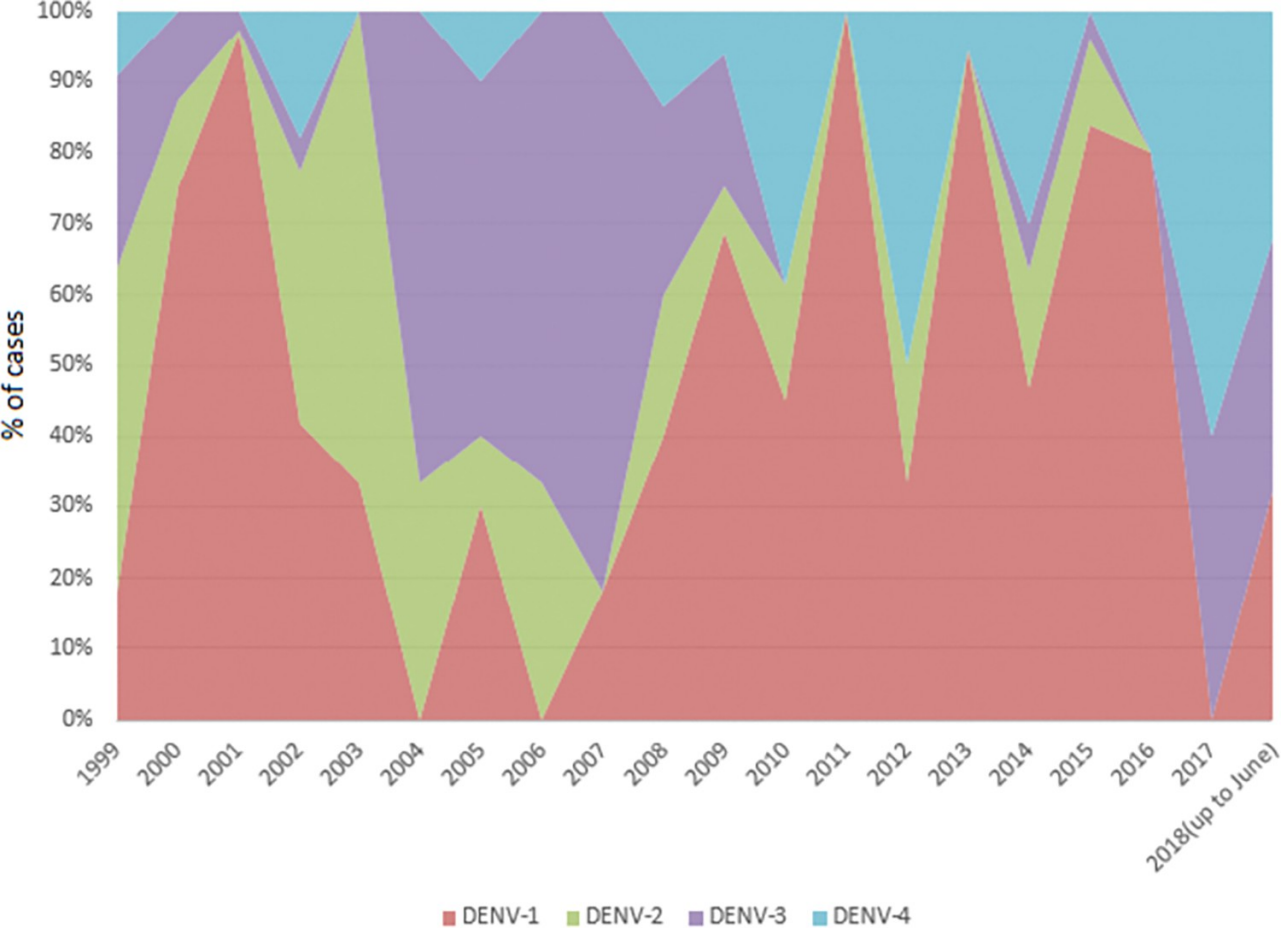
Reported dengue serotypes from 1999 to June 2018.

The available dengue serotype information was from selected hospitals in Yangon, Mandalay, Nay Pyi Taw, Mon and Tanintharyi Regions. The National Health Laboratory requests specimens from cases with severe complications from selected hospitals and serological analysis is done every year in selected Regions [35]. Dengue serotype results for selected Regions were received from the VBDC database from 1999 to June 2018. DENV-1 was the predominant serotype in most years with DENV-2 being predominant in 2003, DENV-3 in 2004–2007 and DENV-4 in 2017 (**Fig 8** and **Table D in S1 Text**).

### Correlation with climatic factors

#### Spearman ρ

**Fig 9:**
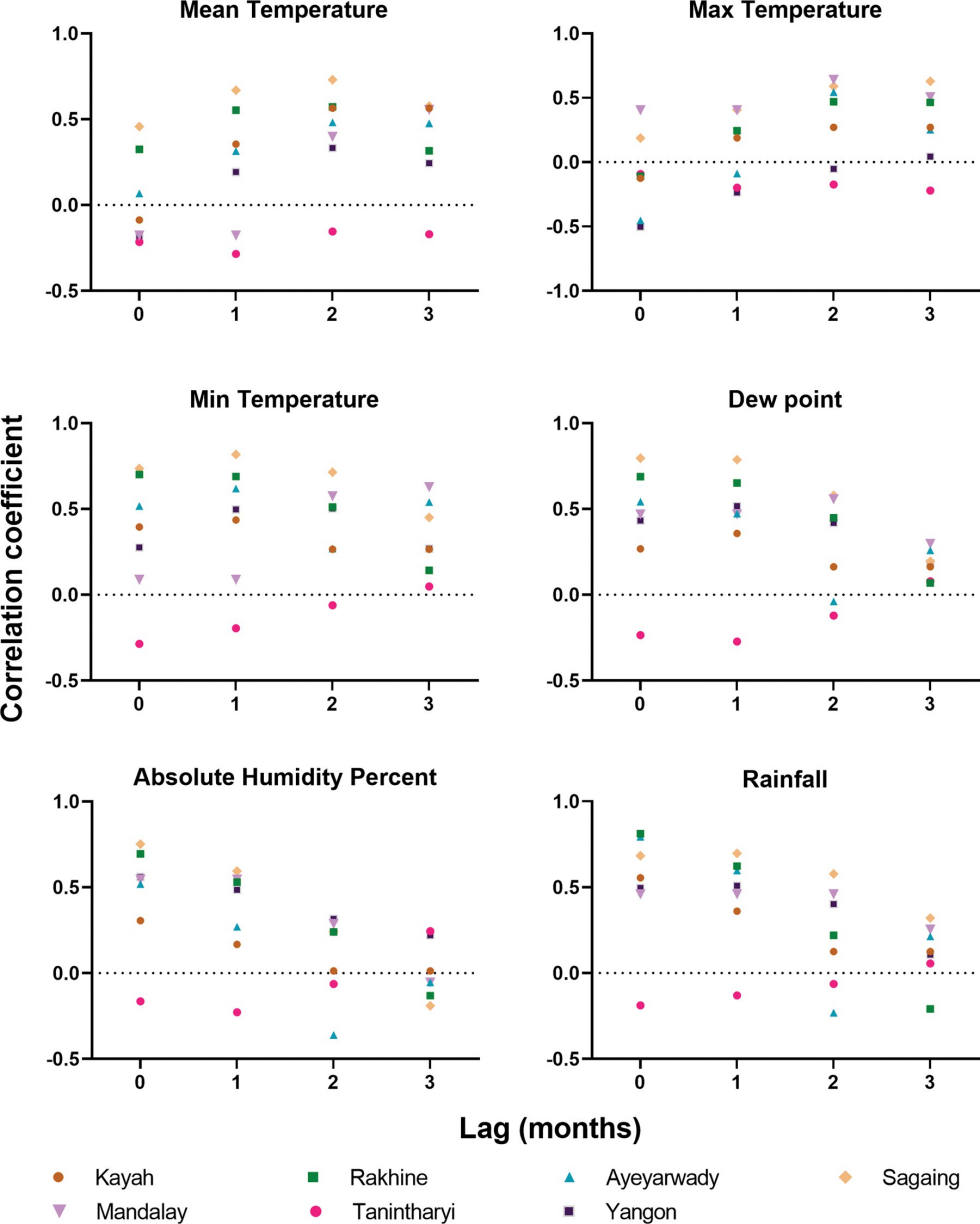
Correlation coefficient of dengue incidence per 100,000 population with various weather variables at different lags for Kachin, Kayah State, Rakhine State, Ayeyarwady Region, Mandalay Region, Sagaing Region, Tanintharyi Region and Yangon Region.

Average monthly rainfall with 0, 1, 2 and 3-month lags had significant positive correlations with dengue incidence in all States and Regions except Tanintharyi Region where none were significant (**Figs 9 andFig E and Table E in S1 Text**). The strength of correlations for rainfall and humidity decreased with increasing lag. Average maximum temperature, minimum temperature and mean temperature had significant mostly positive correlations with dengue incidence with the duration of lag with significance varying between Regions. The strongest correlations for temperature were with minimum temperature at 1 month of lag. Dew point was mostly positively correlated, most strongly at 0–1 months. The correlations with rainfall, temperature and dew point were strongest for Sagaing Region. There was no significant positive correlation with any climate variable at 0, 1 or 2 months for Tanintharyi Region.

#### Univariable DLNM

**Fig 10:**
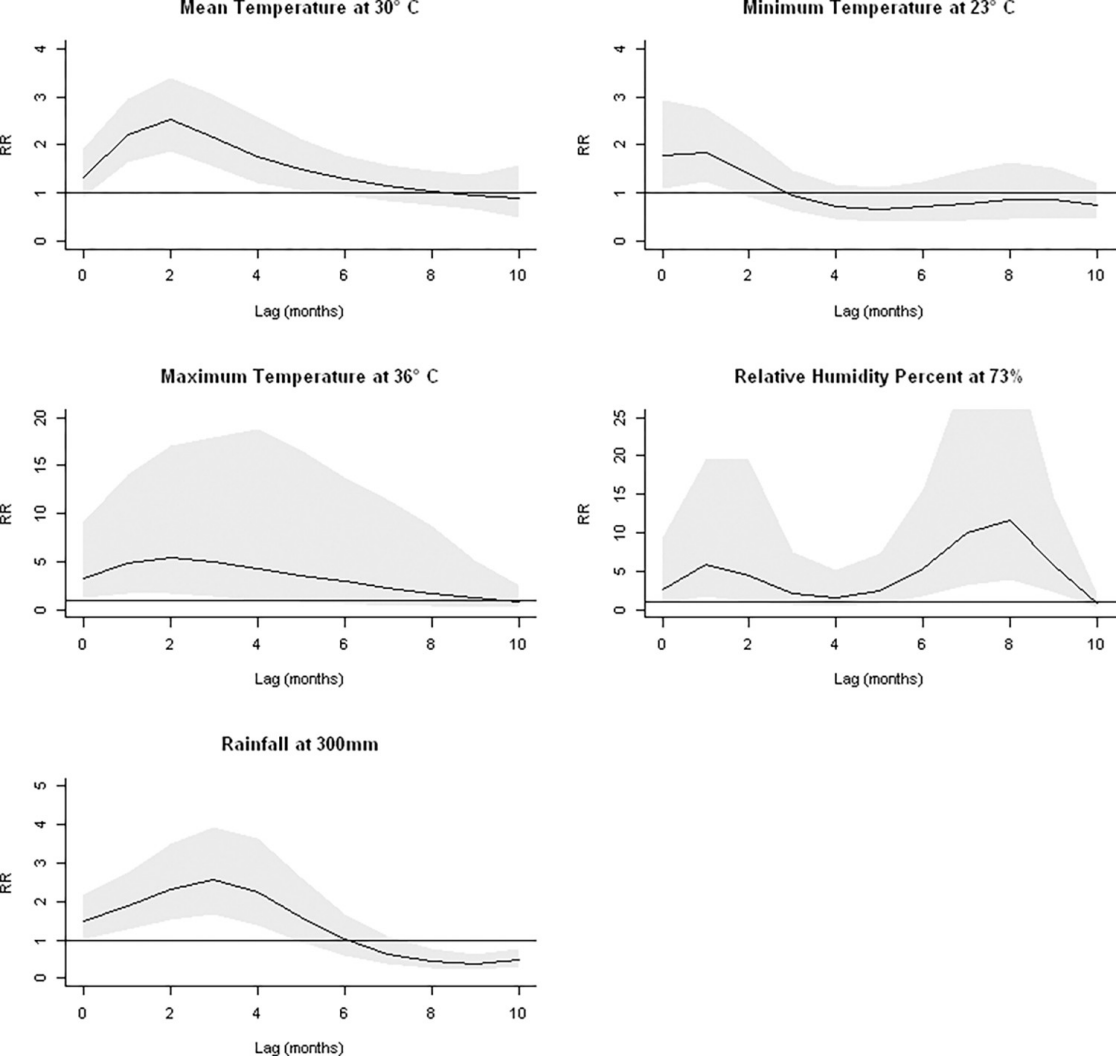
Lag response curves of overall relative risks of each meteorological variable with monthly dengue incidence per 100,000 population in Yangon Region.

In Yangon Region, the RR for association of dengue incidence with minimum temperature peaked at 23°C (RR = 1.84, 95% CI: 1.23–2.77), mean temperature at 30°C (RR = 1.3, 95%CI: 0.8–2.0) and maximum temperature at 37°C (RR = 2.3, CI: 0–12.1) (**Figs 10** and **D in S1 Text**). The elevated risk peaked at 1-month lag for minimum and 3 months for mean and maximum temperatures (**Fig 10**). The risk for relative humidity percent peaked at 73% (RR = 3.5, CI: 0–16.6), declined sharply at 76% and peaked again at 86%. The associated peak risks were at 1-month and 8-month lags respectively. The risk for rainfall peaked at 300mm (RR = 2.5, CI: 1.2–5.0) with peak risk at 2-month lag.

Results of risks for different lags for Ayeyarwady Region, Mandalay Region, Rakhine State, Sagaing Region and Tanintharyi Region are shown in **Figs E to I in S1 Text**. The lag for peak risk and value of relative risk varied between States and Regions across all the weather variables. For Kachin and Kayah States, the univariate model did not converge as there were many months with zero cases.

#### Multi-variable DLNM

**Fig 11:**
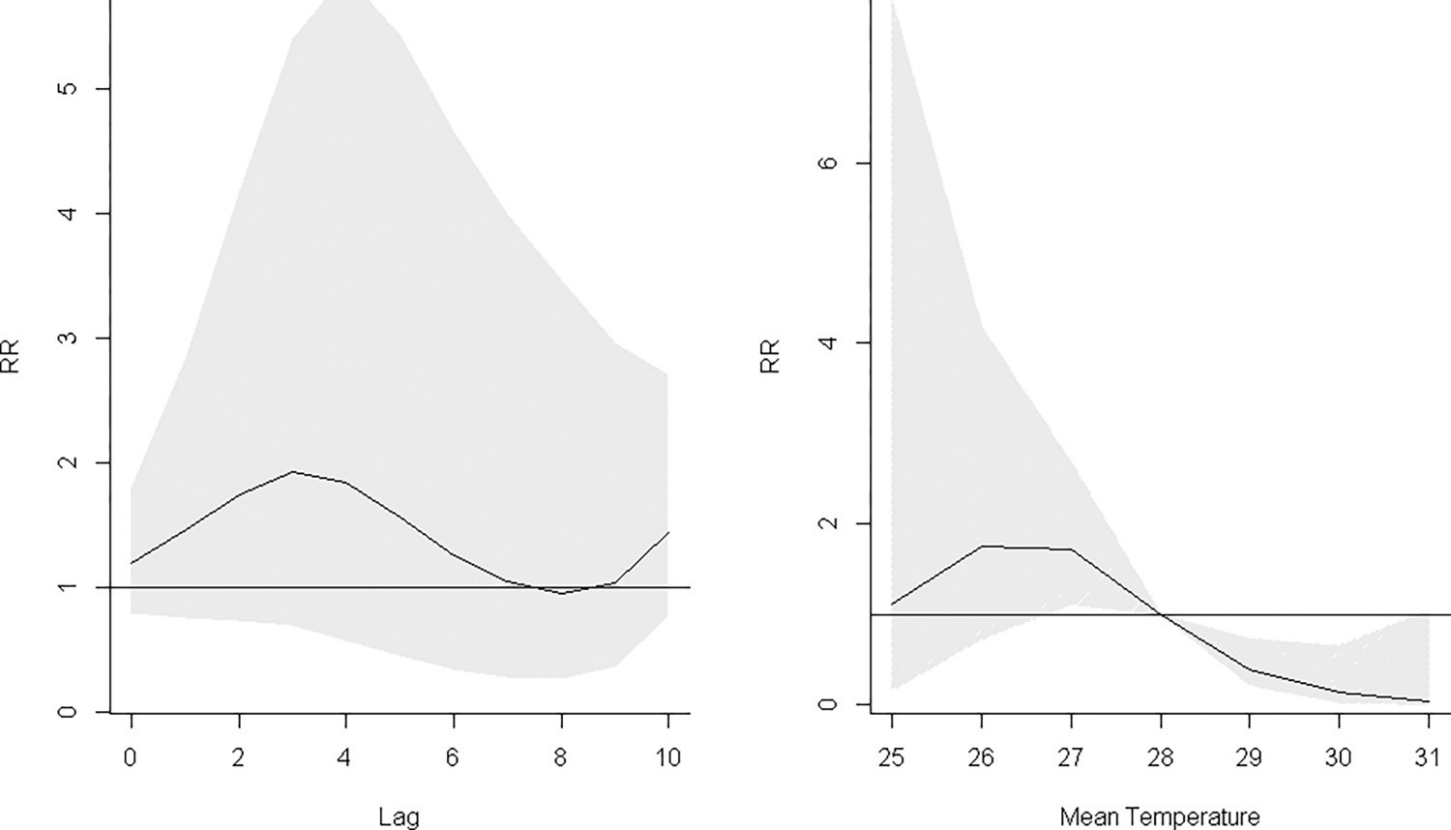
The maximum RR of dengue incidence and mean temperature adjusting for relative humidity and rainfall in Yangon Region. A. Association with temperature at 26°C, centered at 28°C. B. Maximum associated risks at lag 3 months across different temperatures.

This was attempted for all seven States/Regions for which climate data was available: Yangon, Mandalay, Ayeyarwady, Kachin, Kayah, Rakhine, Sagaing and Tanintharyi. Final model results were produced only for Yangon Region, Rakhine State and Tanintharyi Region with results presented below and in **Figs 11**, and **J to O in S1 Text** with correlation matrices in **Tables E to G in S1 Text**. In Kachin and Kayah States, zero inflation was encountered and quasi-likelihood was used to handle the dispersion but parameters were still not well estimated. An alternative could be to use a zero-inflation model which was unfortunately unavailable in the software. In Ayeyarwady and Sagaing Regions, there was high correlation among climatic variables as shown in **Tables H and I in S1 Text** which also caused difficulties in parameter estimation. Thus, in those States/Regions with correlation and excessive zero issues, multivariable analysis was not performed and the Spearman’s coefficient might be more appropriate.

In Yangon Region, the best variable combination for Yangon was mean temperature, rainfall, and relative humidity with QAIC of 953.63. The RR for mean temperature as exposure variable while controlling for rainfall and relative humidity was 1.92 (CI: 0.67–5.41) at 26°C (**Fig 11**). The RR for relative humidity while controlling for mean temperature and rainfall was 2.46 (CI: 0.54–11.29) at 76% (**Fig J in S1 Text**). The RR for rainfall while controlling for mean temperature and relative humidity was 4.61 (CI: 0.46–35.29) at 250mm (**Fig K in S1 Text**). The risk of dengue was highest at mean temperature of 26°C with a 3-month lag (RR = 1.92, CI: 0.69–5.41, **Fig 11**).

In Rakhine State, the best weather variable combination was mean temperature and rainfall (QAIC = 204.42). The RR for mean temperature while controlling for rainfall was 12.59 (CI: 0.73–216.62, **Fig L in S1 Text**) and RR for rainfall controlling for mean temperature is 88.53 (CI: 3.84–2040.27, **Fig M in S1 Text**).

The best weather variable combination for Tanintharyi Region was rainfall and relative humidity (QAIC = 680.37). The maximum RR for rainfall and dengue incidence while controlling for relative humidity was 14.89 (CI: 0.80–278.61) at 700mm (**Fig N in S1 Text**). The risk associated with rainfall continued to exist across all lags up to 9 months. The maximum RR for relative humidity while controlling for rainfall was 7.80 (CI: 0.55–111.16, **Fig O in S1 Text**) at 76%.

### Short-term Dengue incidence forecasting

The best ARIMA model was obtained by comparing and minimizing AIC values from forecasts generated using the *auto*.*arima ()* functions [23]. Forecast accuracies were improved by comparing MSE and R^2^ values in each Region.

**Fig 12:**
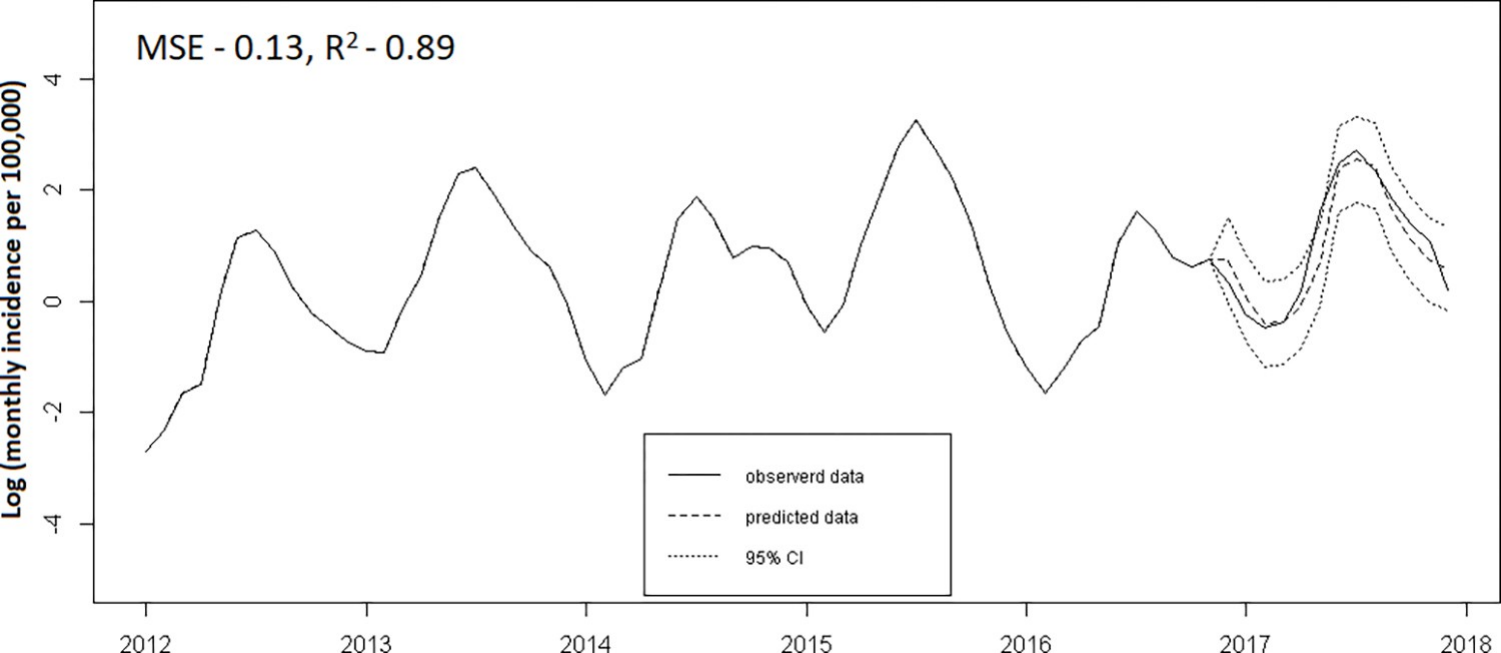
National level dengue forecasts using cases as predictor.

The models with best forecast accuracies were selected after comparing MSE and R^2^ values. At national level, monthly incidence alone was able to predict the overall seasonal pattern **(Figs 12** and **P in S1 Text)**. We compared different model specifications summarized in **Table J in S1 Text**. We included the seasonal component alone in models 2.4, 3.3 and 4.3 and the results were worse than using incidence data or incidence with weather data suggesting it didn’t contribute to short-term prediction. This may be because the seasonal information was already absorbed in the incidence and climatic covariates in the model. In addition, the lagged correlation also suggests short term correlation between case lags.

**Fig 13:**
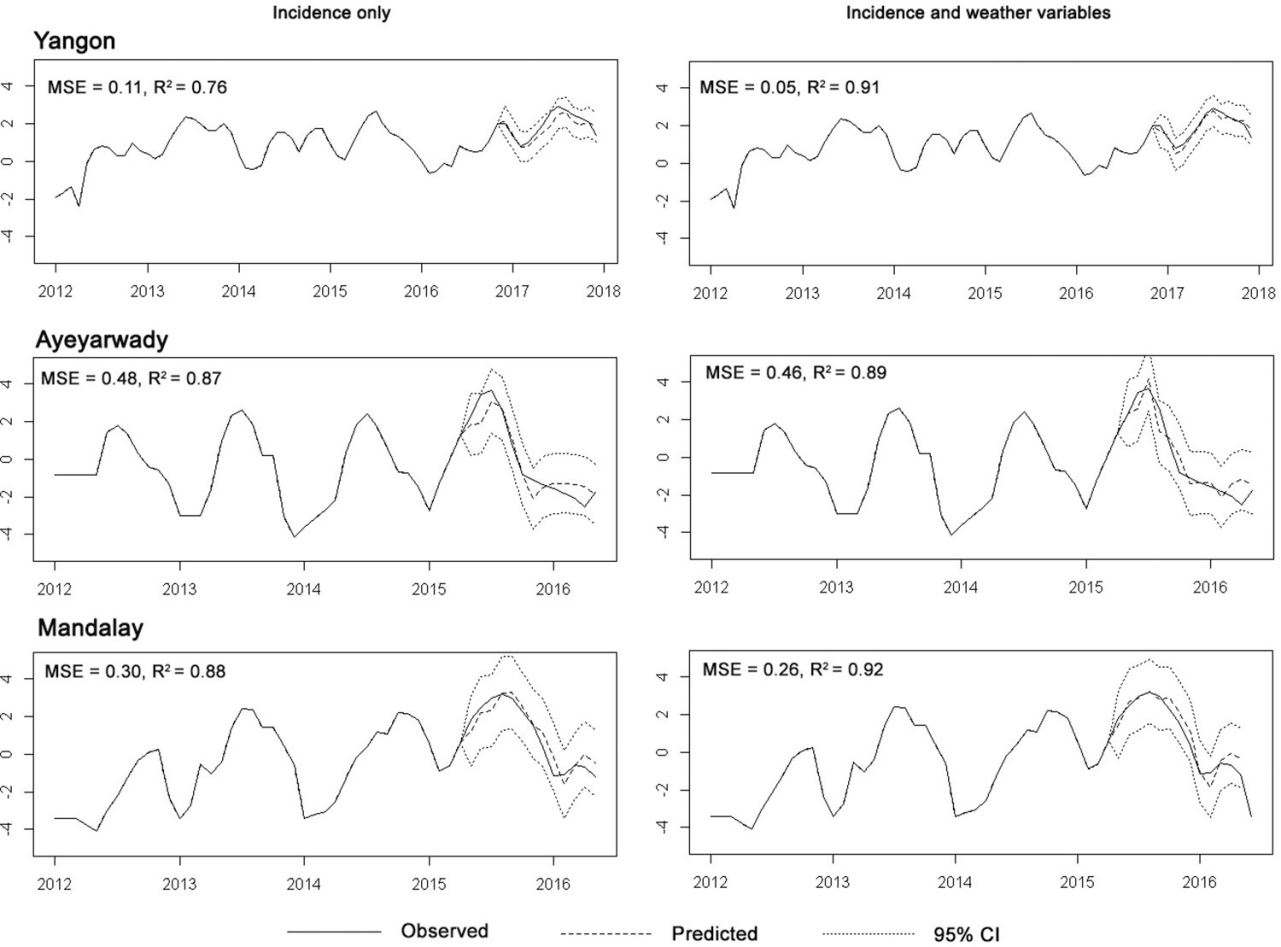
Comparisons of dengue forecasts between incidence and incidence plus weather variables showing the best performing combination of weather variables for each State/Region. Lines for observed incidence, predicted incidence and 95% confidence interval for predicted incidence are shown.

Forecasts for Yangon, Ayeyarwady and Mandalay Regions were all improved by including weather variables, although the magnitude of this improvement was small (**Fig 13** and Fig **Q and Table K in S1 Text**). Monthly percent humidity and dew point temperature at one-month lags and rainfall and minimum temperature at 3-month lags, were the best predictor climate variables for monthly dengue incidence in Yangon Region. Improvements in model performance with climate for the other Regions were more modest. In Ayeyarwady Region, percent humidity at one-month lag, rainfall at 0-month lag and dew point temperature at 1-month lag were the best predictors and in Mandalay Region, dew point temperature at 3-month lag, rainfall at 0-month lag and minimum temperature at 1-month lag were best predictor variables. Changing the duration of the lags made only a small difference to model performance.

## Discussion

From 2012 to 2017 in Myanmar there was a strong seasonal pattern of dengue incidence with timing of peak incidence and outbreak years varying between different States and Regions. Dengue was reported in more townships over time despite significant drops in cases in non-outbreak years in 2012, 2014 and 2016. With development of the public health surveillance system over time in Myanmar, it seems more likely that this change is due to improvements in surveillance and reporting and better access to health services in more remote parts of the country than an actual spread of dengue over time [36]. There were no long-term trends in the climate variables in our data so they were unlikely to contribute to the increasing spatial extent. Strengthening surveillance has included support from WHO from 2014 to 2018 for better data collection by health facilities and at township level, strengthening the Health Management Information System, improving access to care, improving coordination and resource mobilization, and rapid response to disease outbreaks. [36] Although incidence rates were highest in Mon State, particularly Mawlamyine, Mudon, Paung and Kyaikmaraw townships, Yangon Region had the highest absolute numbers of cases and deaths, likely because it is by far the largest city in the country. Similar to previous observations [35], numbers of deaths reflected the numbers of cases and the majority of the mortality was in patients diagnosed with dengue severity grade-4 (DSS).

There was a roughly even split of cases between urban and rural areas. The urban cases were mostly in Yangon and Mandalay Regions, which have large urban populations, with cases and deaths in the other States and Regions being reported from rural areas. With a split of the general population into 30% urban and 70% rural, this suggests a relative excess of cases in urban areas, as has been reported elsewhere [37]. More recent evidence from Viet Nam suggests lower population densities have higher potential for dengue outbreaks although absolute numbers of cases in rural areas would still be lower than urban areas [38]. To meet the national goals for dengue control [18] will require concerted efforts across much of the country engaging populations in both urban and rural areas.

There were no stable spatial pattern of dengue incidence at subnational levels and it was observed that outbreak frequency was higher compared to that reported for the previous ten years [18]. The age distribution of cases and deaths remained the same over time among children with the 5–9 year age group having the most morbidity and mortality. In a previous study in Myanmar [35], the highest risk age groups for death from dengue were infants and age group >15 years. Possible reasons for this difference are changing availability of data or reporting practices over time. VBDC only released age group breakdown of cases and fatalities country wide from 2016 onward. In 2015, there was an unusual increase in the occurrence of adult dengue cases for patients admitted at Yangon General Hospital [39] and in our study, we found that the incidence of dengue in adults increased 6-fold from 2012 to 2017. The age distribution overall had a relative lack of adults compared to other countries with similar epidemiology [40], although this was increasing over time. This low incidence in adults may be because of higher transmission with higher attack rates for symptomatic infection in children, potentially compounded by under-reporting in older age groups. Another possible explanation is reporting bias of physicians caring for adults who do not sufficiently suspect or report dengue; with the data suggesting a gradual improvement in this practice.

In our dataset, there was variation in the most prevalent serotype over time, although all four were present throughout. DENV-1 was the most dominant serotype, and DENV-2, DENV-3, DEN-4 were roughly equally distributed. There was very limited sample size with the data being from routine surveillance and serotype data from research studies not being included in the VBDC database thus limiting the possibility of making any inferences about relationship to outbreaks and trends over time. Research studies looking at serotype patterns in Myanmar [35] described larger sample sizes than in the routine surveillance database and identified higher numbers of infections of all four serotypes from 2013 to 2015. Studies of dengue outbreaks in 2013 and 2015 found that there was no cross correlation among serotypes with DENV-1 being predominant in 2013 [41] and all 4 serotypes being present in the 2015 [35] outbreak, both of which were consistent with our data. In the most recent year in our serotype data, 2018, DENV-3 was the predominant serotype. More widespread collection of serotype data from all DHF cases would be more informative.

Average mean temperature, minimum temperature and maximum temperature at different lags were positively correlated with dengue incidence [6–9]. The survival rate of *Aedes* mosquitoes is related to increases in temperature and their ability to travel long distances travelling with people [42–44], resulting in dengue spreading to new areas. Historical data from the Climate Change Network portal of the World Bank, show the ten-year average temperature in Yangon to be gradually increasing, from average 22.71°C in 1901–1910, to 23.28°C in 2000–2010 and 23.38° C in 2010–2016. Minimum temperature was consistently positively correlated with incidence whereas the relationship for maximum temperature was weaker and sometimes negatively correlated for some States/Regions (Yangon and Ayerawaddy). This may be a reflection of there being an ideal temperature for breeding of the mosquito vectors of 17°C to 35°C [45, 46] with increases in maximum temperature beyond that range reducing the chance of vector survival. The States/Regions with negative correlations with maximum temperature had more days per year above this range than those with all positive correlations. In Tanintharyi Region, the correlations were much weaker than the others, suggesting another as yet unidentified factor is strongly driving transmission in this area.

There was a strong seasonal pattern of dengue incidence in Myanmar with the timing of the season correlated with peaks in rainfall. Percent humidity and DEW point are high when rainfall and temperature are high and these conditions enhance breeding and survival of the vector population [47]. Other studies have also confirmed that (mean, maximum and minimum) temperature was significantly associated with dengue incidence at a lag of 0 to 3 months e.g. in Cambodia [25] and Singapore [48]. Rainfall and Humidity were positively correlated with dengue incidence and studies suggest that global increases in temperature, rainfall and humidity are possible factors behind the increase in dengue epidemics [42, 49, 50]. In the present study, we found that peaks in dengue generally coincided with and sometimes preceded peaks in rainfall. This may be because excess rainfall can wash away mosquito breeding sites and cause a decrease in dengue transmission [51].

The models with best forecast accuracies differed by location. Predictions for the whole country and for Yangon Region were close to observed data and had narrower confidence intervals than those of other Regions. Time series analysis with an ARIMA model closely predicted the trend and the peak months. Incorporating weather variables improved the predictive power of the ARIMA model particularly in Yangon, with more modest improvements in Ayeyarwady and Mandalay Regions. However, sufficiently up to date climate station data is currently difficult for the National Dengue Programme to access in time to use in a model and there was no climate station data available in the publicly available datasets for many areas, including other States/Regions. There is potential for remote sensing data to substitute with further research to quantify the strength of correlation but such data is technically challenging to process and not currently feasible for the dengue control programme to use on a short timescale needed for guiding interventions ahead of time. Overall, performance improvements on adding climate data were small and the additional effort may not be warranted as the practical impact of marginal improvements in predictive power on planning dengue control activities is likely to be minimal. The results show ARIMA using historical incidence data is a simple and effective method for predicting future dengue incidence and is useful for rapidly analysing surveillance data as previously described [52] including in similar studies in Colombia [29], Bangladesh [53], Brazil [54] and Bhutan [55]. This prediction method is better than using historical seasonality alone as the timing and height of peaks in incidence varies between different parts of the country and changes from year to year.

The selected method for Myanmar of one month ahead prediction balances providing sufficient warning for the control programme to implement interventions to reduce transmission against the loss of prediction accuracy seen over longer timescales thus minimizing the occurrence of false alarms. These interventions may include mosquito habitat reduction by reducing standing water sources for which a public health education campaign could be triggered by the early warning from a prediction model. An alternative could be anticipatory insecticide fogging of areas with high predicted incidence. With further development and testing, this method has potential to enhance preparedness by short-term forecasting of dengue outbreaks in Myanmar giving decision makers some advance warning and more time to plan control measures, public health messaging and allocation of additional resources for healthcare. This would be an improvement on the current system of reacting to changes in reported case numbers. Further research is needed to identify if there are other variables which could improve the predictions whilst also being readily available to the government for use in real-time including in other countries. Improving the certainty of predictions over longer timescales may be particularly useful, as demonstrated using cellphone-derived mobility data in Thailand. [9].

There are some limitations in this study. We used aggregated routine surveillance data collected by the Ministry of Health from cases admitted to hospital. It was not possible to thoroughly assess the quality of the data as there was no comparator dataset available and no information on completeness of reporting. Basic checks on completeness and consistency revealed no issues. Dengue case reporting in Myanmar is health facility-based and the data received were already aggregated at township level. This also limited the data quality checks that could be done and constrained the spatial scale of the analysis. The township level data was only available aggregated by year and State/Region by month which further limited the possible analyses. During the period of the study, there have been efforts to improve the communicable disease surveillance system in Myanmar, including by WHO [36], but it was not possible to quantify the impact of this on the consistency of data over time. Cases of suspected dengue in Myanmar are not routinely tested and even where available this test result is not recorded in the central surveillance database. Thus, most cases are diagnosed using WHO clinical criteria alone. We have shown previously in a systematic review that the sensitivity of the 1997 WHO case definition is 93% with a specificity of 29%. Many of the cases reported as dengue may therefore have had a different diagnosis [56]. The data used in this study was DHF cases only as that is what is collected by the government. As the case definition for DHF requires haemorrhagic manifestations, and these are uncommon in other febrile illnesses in Myanmar, the specificity is likely to be higher. Additionally, many people with dengue do not attend health services and are therefore not reported, thus leading to an under-estimate of disease which is likely to be greater in areas with poorer access to health services. Thus, under-served populations and those living in remote areas are more likely to be under-represented in the data. It was not possible to quantify the impact of changes in surveillance on the data over time. Assuming the under and over-reporting fractions were similar over the study period, the methods and overall space-time incidence trend should still be valid. If surveillance substantially improved, this may result in an increase in completeness of case data with better ability to accurately predict short-term trends. We are not aware of substantial changes to these limitations since 2017, although an updated analysis with data from 2018–2019 may be informative but this data was not available to the study team. Five states (Bago, Magway, Mon, Kayin and Shan) were omitted from the correlation and predictive analysis using climate data due to there being no climate stations in those states. Four of these (Bago, Magway, Mon and Kayin) are in the tropical climate zone with similar climates to Yangon and Ayeyarwady. The other (Shan) is in the temperate zone with similar climate to Kachin. Thus, if climate data were available, it could be expected to have a similar modest impact on predictive ability of the models. Further studies are needed to quantify the true burden of dengue in Myanmar and confirm which population groups are most affected, as well as to identify modifiable risk factors to reduce transmission. Future research might also focus on predicting where future outbreaks are likely to occur. Climate change has also been predicted to contribute to an increase in dengue incidence over longer timescales [57–59] and further studies to understand this would be important for Dengue Control Programmes to plan future interventions.

In conclusion, analysis of routine surveillance data on mostly hospitalised cases with dengue in Myanmar was able to show, despite limitations and biases, a clear seasonal pattern associated with rainfall with biennial peaks in incidence and an increase in the geographic distribution of cases over time. The timing of outbreaks varied between different parts of the country. Using a simple model, it was possible to make accurate short-term predictions of incidence which have potential to guide planning of upcoming dengue control activities.

## Supporting information

S1 RECORD Checklist The RECORD statement–checklist of items, extended from the STROBE statement, that should be reported in observational studies using routinely collected health data. (PDF)

S1 Text **Fig A in S1 Text.** Proportion of dengue cases in urban and rural areas. **Fig B in S1 Text.** State/Region level monthly dengue cases and number of deaths. **Fig C in S1 Text.** Dengue severity grades by States and Regions in 2016 and 2017. Source and terms of use for shapefile: https://geonode.themimu.info/layers/geonode%3Ammr_polbnda_adm1_250k_mimu. **Fig D in S1 Text.** Overall association of univariate analysis with minimum temperature, mean temperature, maximum temperature, rainfall and relative humidity percent with dengue incidence in Yangon Region. **Fig E in S1 Text.** Lag response curves of overall relative risks of each meteorological variable with monthly dengue incidence per 100,000 population for Ayerarwady Region. **Fig F in S1 Text**. Lag response curves of overall relative risks of each meteorological variable with monthly dengue incidence per 100,000 population for Mandalay Region. **Fig G in S1 Text.** Lag response curves of overall relative risks of each meteorological variable with monthly dengue incidence per 100,000 population for Rakhine State. **Fig H in S1 Text.** Lag response curves of overall relative risks of each meteorological variable with monthly dengue incidence per 100,000 population for Sagaing Region. **Fig I in S1 Text.** Lag response curves of overall relative risks of each meteorological variable with monthly dengue incidence per 100,000 population for Thanintharyi Region. **Fig J in S1 Text**. Results of multivariable analysis of maximum RR of dengue incidence and climate for relative humidity percent adjusting for mean temperature and rainfall and in Yangon Region. A. Lag response curve, association with relative humidity at 76%, centered at 82.5%. B. Maximum associated risks at lag 6 months across different humidity levels. **Fig K in S1 Text**. Results of multivariable analysis of maximum RR of dengue incidence and climate for rainfall adjusting for mean temperature and relative humidity in Yangon Region. A. Lag response curve, association with rainfall at 250mm, centered at 500mm. B. Maximum associated risks at lag 8 months across different rainfall levels. **Fig L in S1 Text**. Results of multivariable analysis of maximum RR of dengue incidence and climate for mean temperature adjusting for rainfall in Rakhine State. A. Lag response curve, association with mean temperature at 29°C, centered at 27°C. B. Maximum associated risks at lag 8 months across different mean temperatures. **Fig M in S1 Text**. Results of multivariable analysis of maximum RR of dengue incidence and climate for rainfall adjusting for mean temperature in Rakhine State. A. Lag response curve, association with rainfall at 350mm, centered at 1250mm. B. Maximum associated risks at lag 10 months across different rainfall levels. **Fig N in S1 Text**. Results of multivariable analysis of maximum RR of dengue incidence and climate for rainfall adjusting for relative humidity in Tanintharyi Region. A. Lag response curve, association with rainfall at 700mm, centered at 400mm. B. Maximum associated risks at lag 8 months across different rainfall levels. **Fig O in S1 Text**. Results of multivariable analysis of maximum RR of dengue incidence and climate for relative humidity adjusting for rainfall in Tanintharyi Region. A. Lag response curve, association with relative humidity at 76%, centered at 80%. B. Maximum associated risks at lag 9 months across different humidity levels. **Fig P in S1 Text.** Residuals of incidence prediction results using ARIMA model without (left) and with (right) weather variables for the whole country. A. Residuals of the model by month, B. Autocorrelation plot (ACF), C. Histograms of the residuals. **Fig Q in S1 Text.** Residuals of incidence prediction results using ARIMA model without (left) and with (right) weather variables for Yangon, Mandalay, Ayeyarwady. A. Residuals of the model by month, B. Autocorrelation plot (ACF), C. Histograms of the residuals. **Table A in S1 Text.** Annual dengue incidence per 100,000 population by State and Region. **Table B in S1 Text.** Annual dengue case fatality rates by State and Region. **Table C in S1 Text.** Reported dengue serotypes from 1999 to June 2018. **Table D in S1 Text.** Correlation of dengue incidence per 100,000 population with different weather variables in Kachin, Kayah, Ayeyarwady, Mandalay, Sagaing, Tanintharyi and Yangon Regions. **Table E in S1 Text.** Correlation matrix of climate variables for Yangon Region. **Table F in S1 Text.** Correlation matrix of climate variables for Rakhine State. **Table G in S1 Text.** Correlation matrix of climate variables for Thanintharyi Region **Table H in S1 Text.** Correlation matrix of climate variables for Ayeyarwady Region. **Table I in S1 Text.** Correlation matrix of climate variables for Sagaing Region. **Table J in S1 Text**. Comparisons of different regions’ ARIMA model order with and without covariates for the whole country, and for Yangon, Mandalay and Ayeyarwady Regions. **Table K in S1 Text**. Prediction comparisons of observed and predicted data with the best model and seasonality alone (first seasonal autoregressive and difference order) for the whole country and Mandalay Region, Mon State, Yangon Region and Ayeyarwady Region.
(DOCX)
