# Supplementary material for: Dengue in Myanmar: Spatiotemporal epidemiology, association with climate and short-term prediction
Source: PLoS Negl Trop Dis. 2023 Jun 5;17(6):e0011331. doi: 10.1371/journal.pntd.0011331 (PMC10270578; doi:10.1371/journal.pntd.0011331)

**Supporting Information**


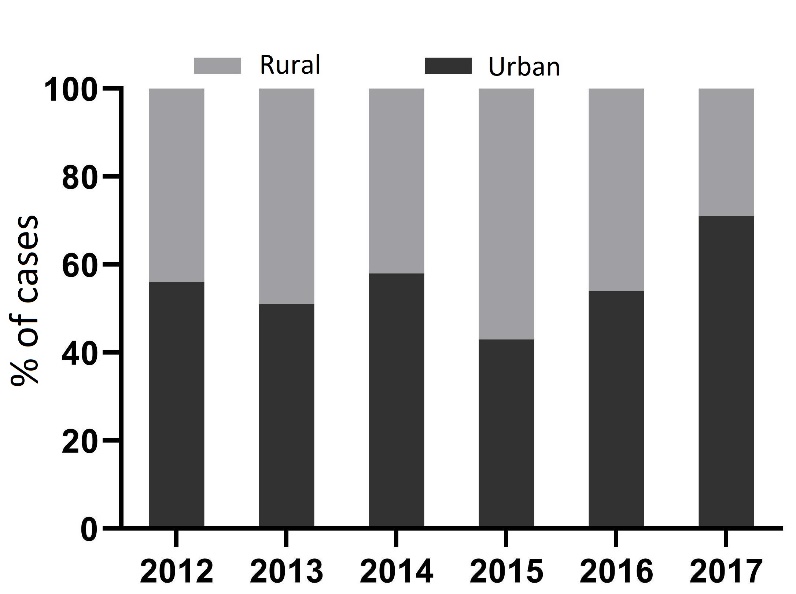


**Fig A**. Proportion of dengue cases in urban and rural areas.


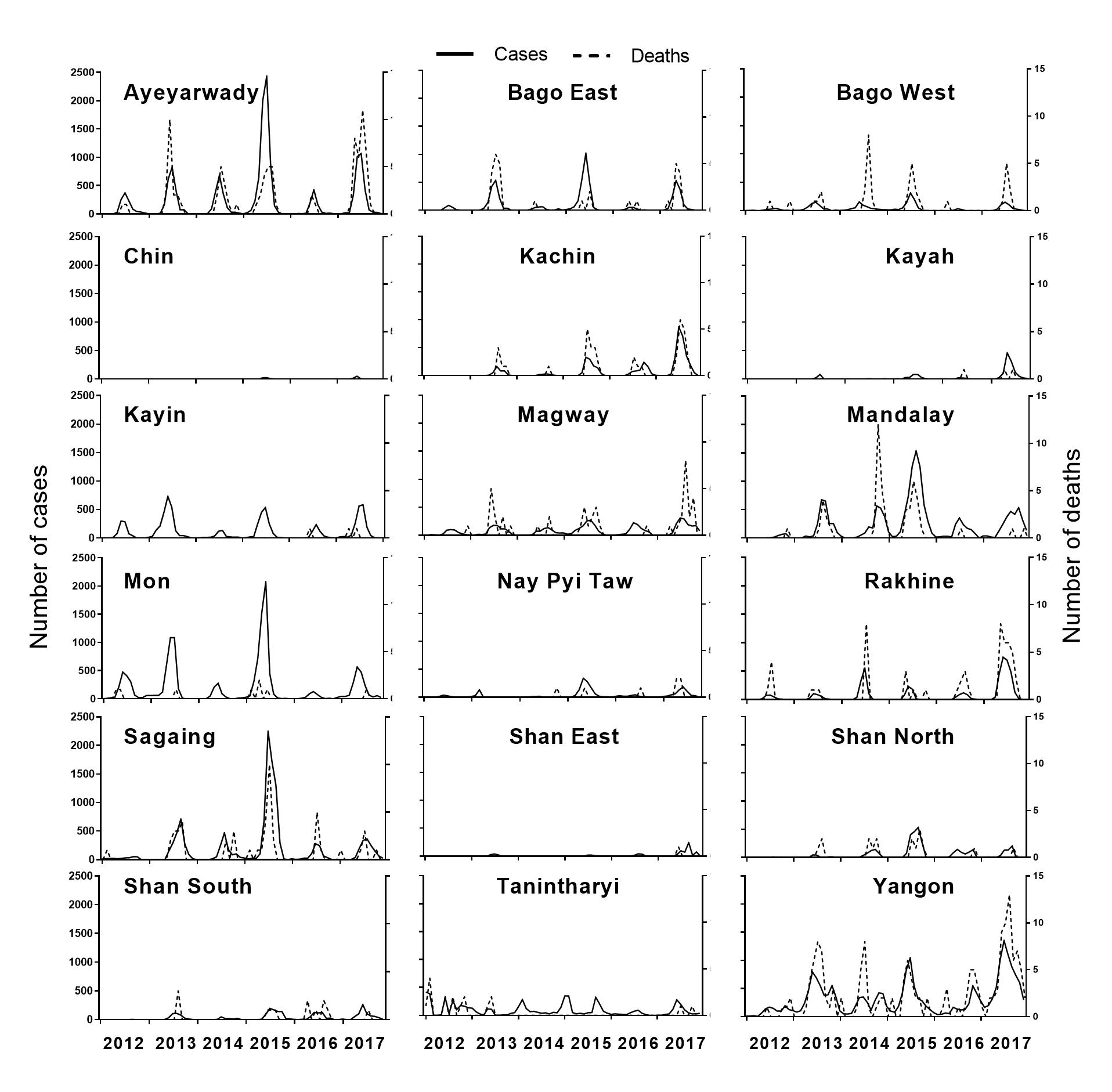


**Fig B**. State/Region level monthly dengue cases and number of deaths.


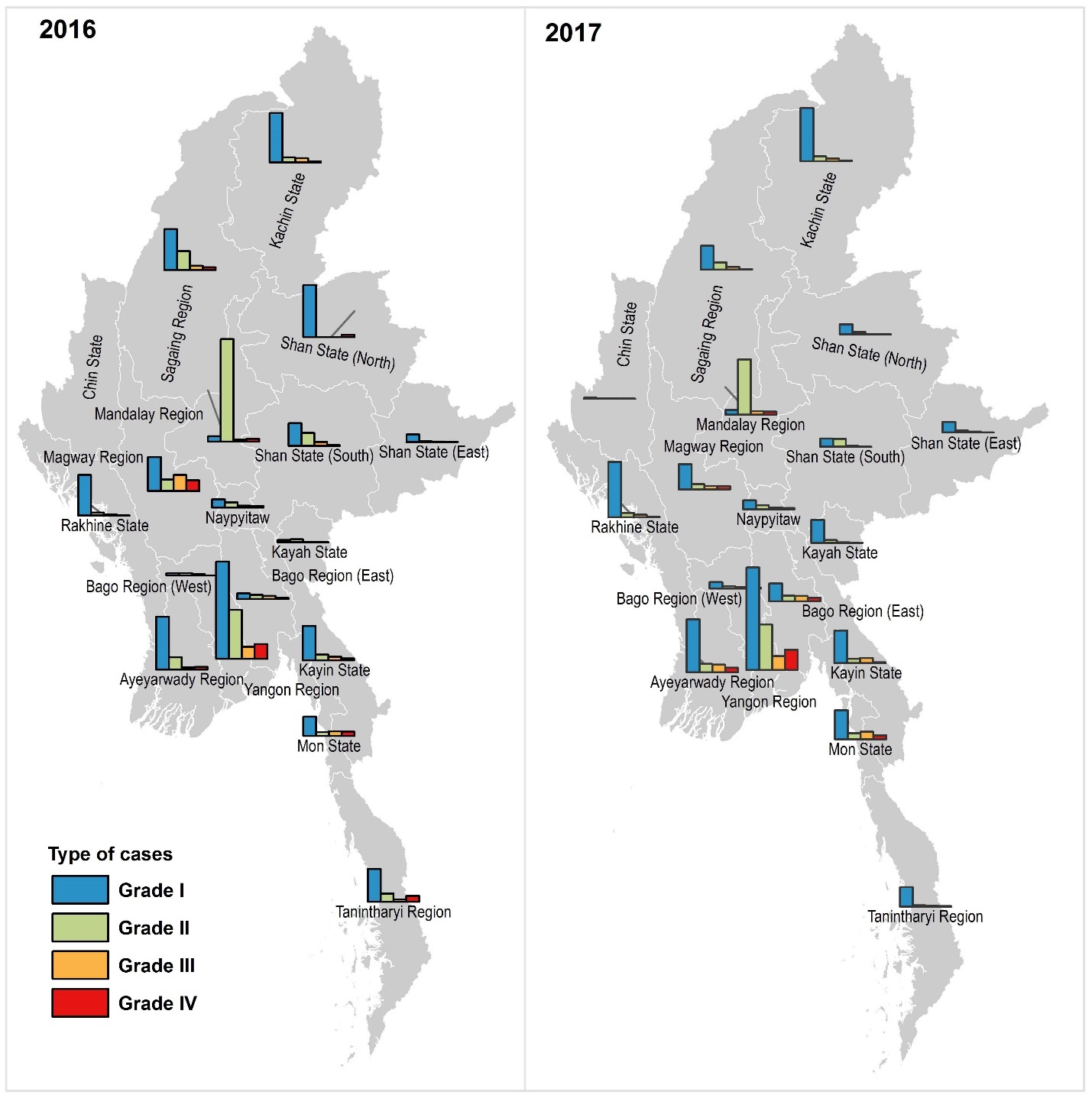


**Fig C**. Dengue severity grades by States and Regions in 2016 and 2017. Source and terms of use for shapefile: <https://geonode.themimu.info/layers/geonode%3Ammr_polbnda_adm1_250k_mimu>.


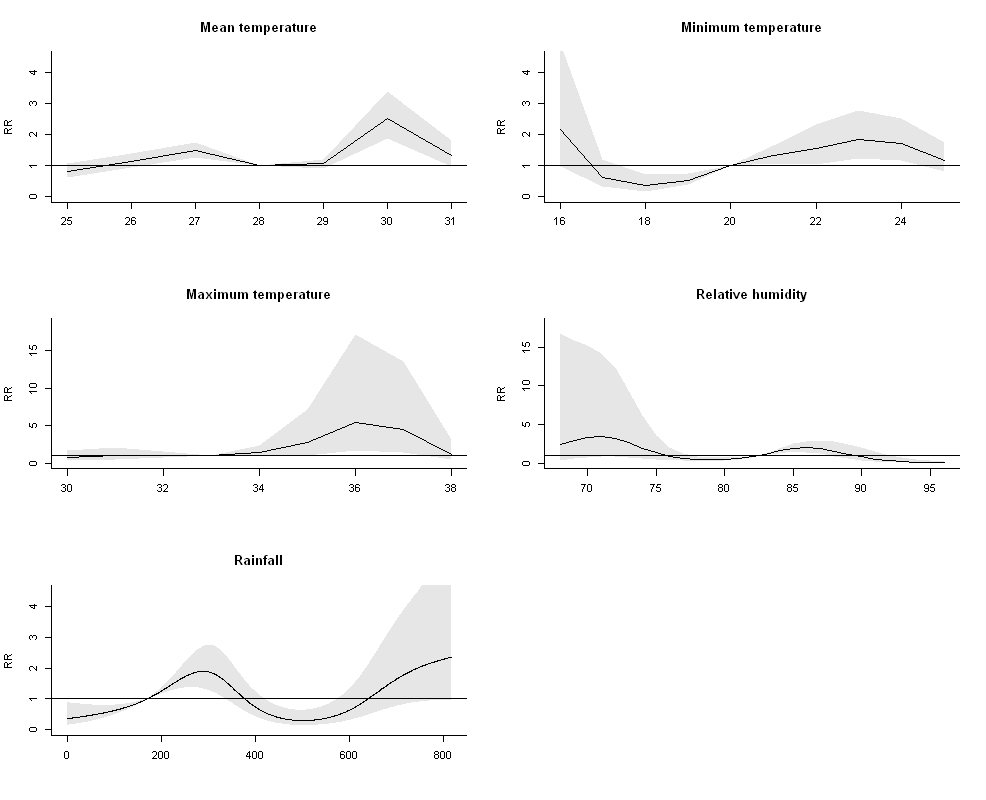


**Fi****g D**. Overall association of univariate analysis with minimum temperature, mean temperature, maximum temperature, rainfall and relative humidity percent with dengue incidence in Yangon Region.

**
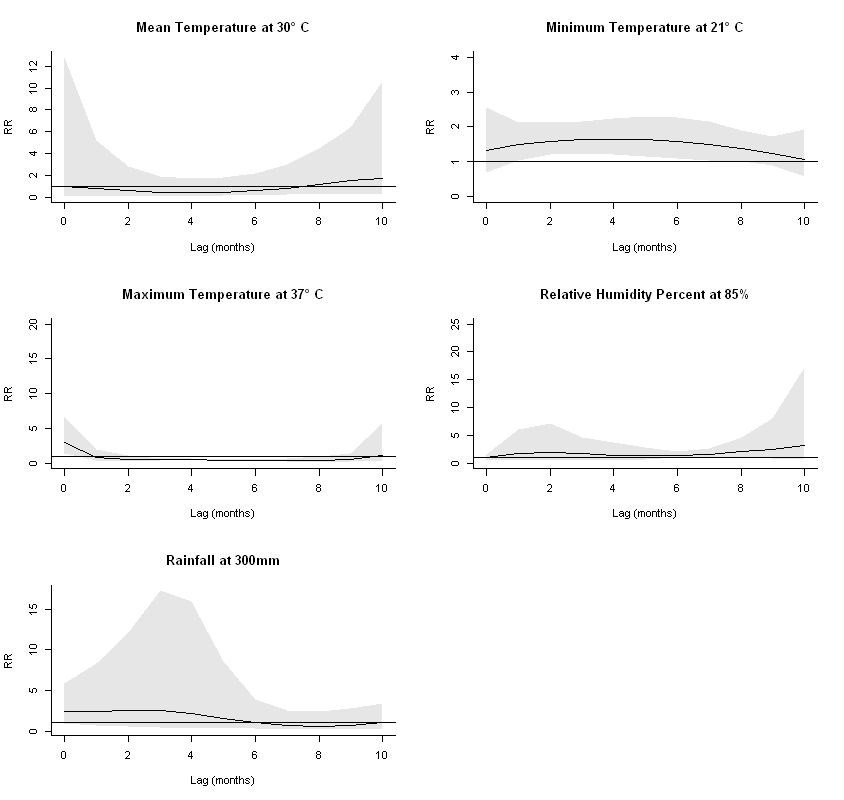
**

**Fig E.** Lag response curves of overall relative risks of each meteorological variable with monthly dengue incidence per 100,000 population for Ayerarwady Region.

**
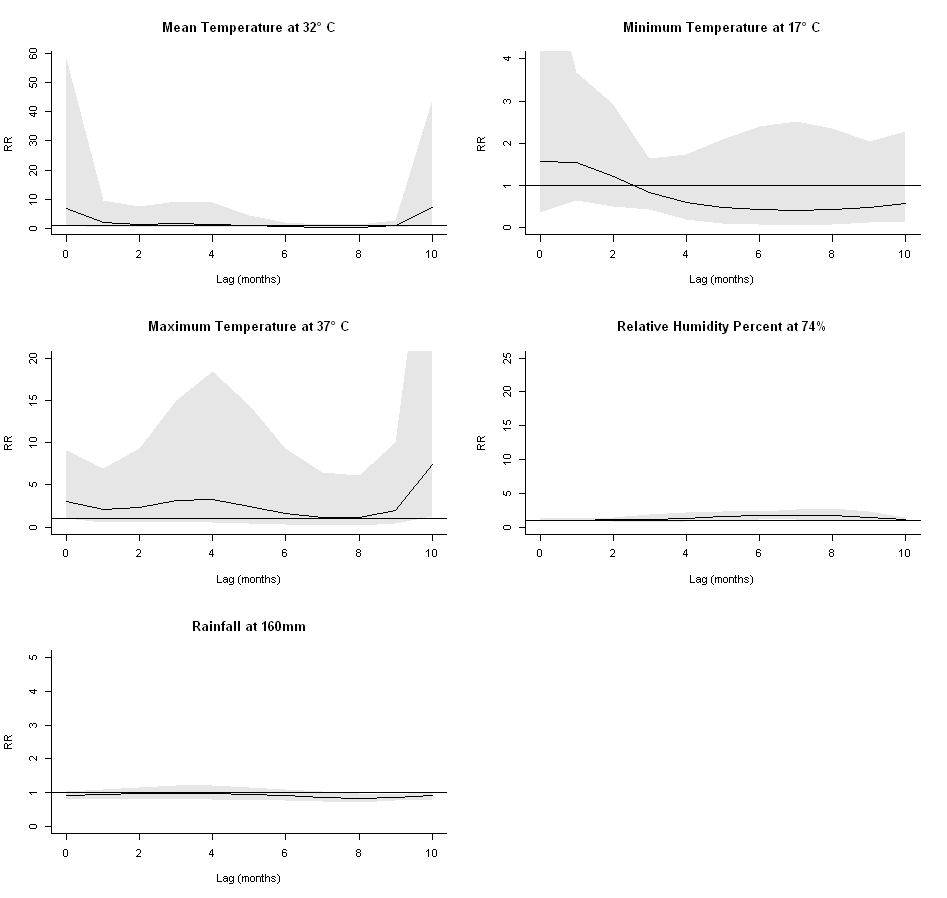
**

**Fig F.** Lag response curves of overall relative risks of each meteorological variable with monthly dengue incidence per 100,000 population for Mandalay Region.

**
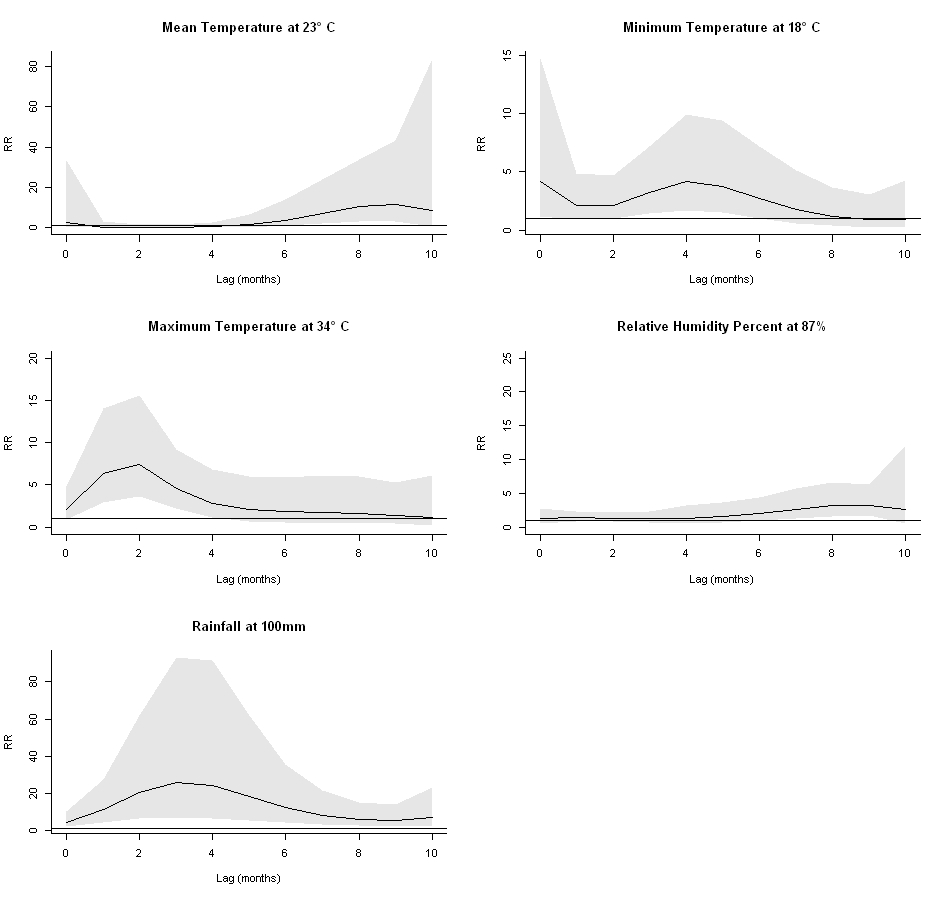
**

**Fig G.** Lag response curves of overall relative risks of each meteorological variable with monthly dengue incidence per 100,000 population for Rakhine State.

**
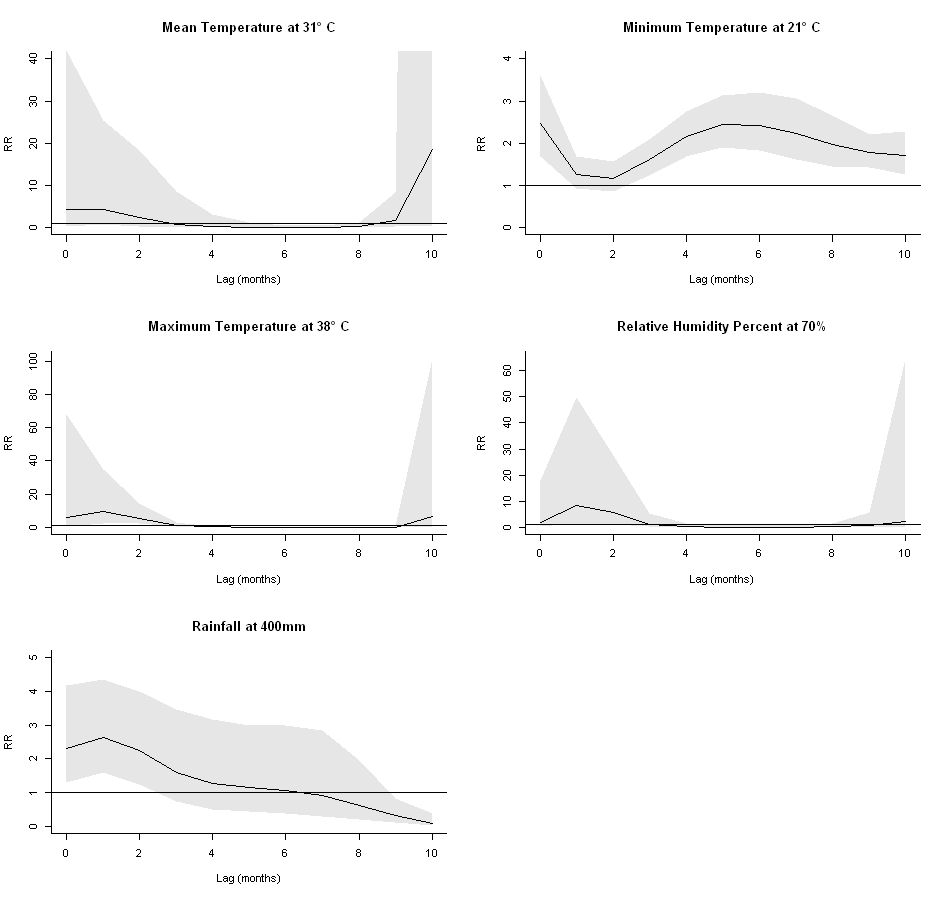
**

**Fig H.** Lag response curves of overall relative risks of each meteorological variable with monthly dengue incidence per 100,000 population for Sagaing Region.

**
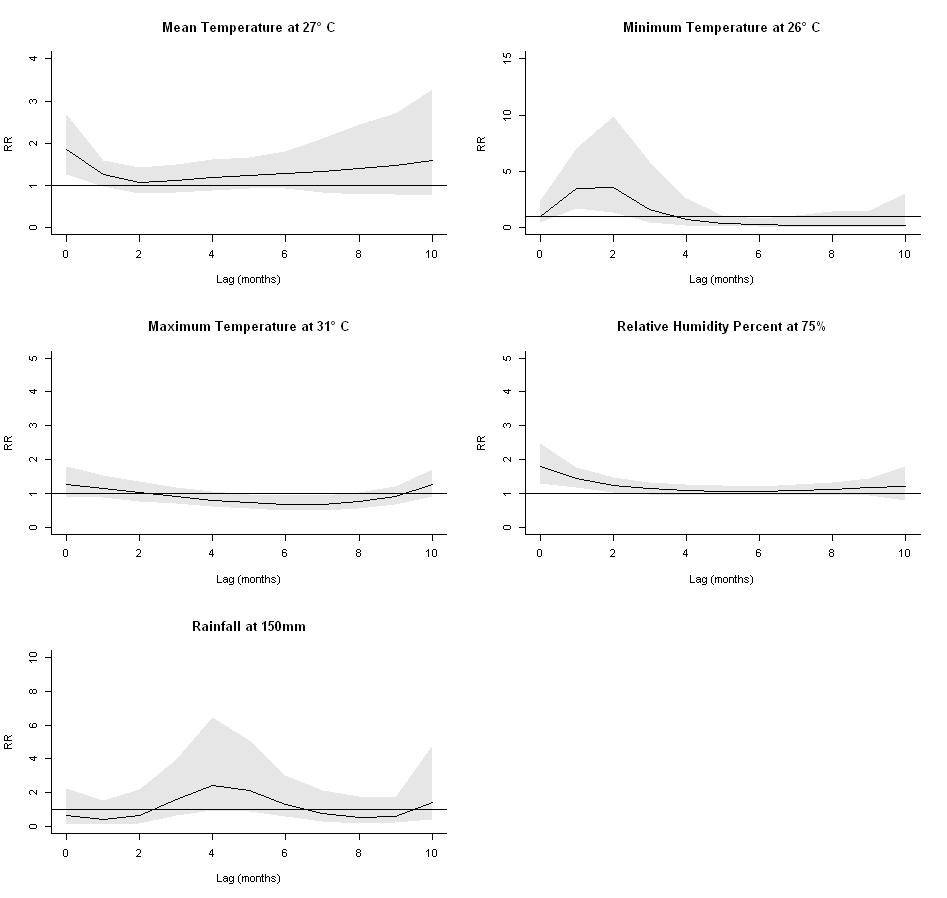
**

**Fig I.** Lag response curves of overall relative risks of each meteorological variable with monthly dengue incidence per 100,000 population for Thanintharyi Region.


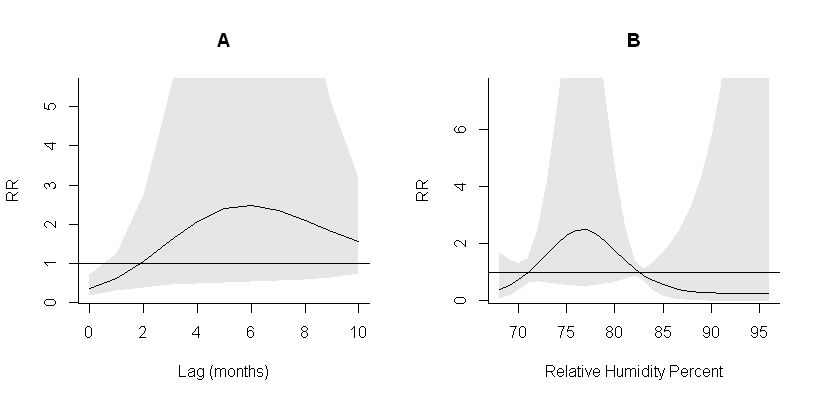


**Fig J**. Results of multivariable analysis of maximum RR of dengue incidence and climate for relative humidity percent adjusting for mean temperature and rainfall and in Yangon Region. A. Lag response curve, association with relative humidity at 76%, centered at 82.5%. B. Maximum associated risks at lag 6 months across different humidity levels.
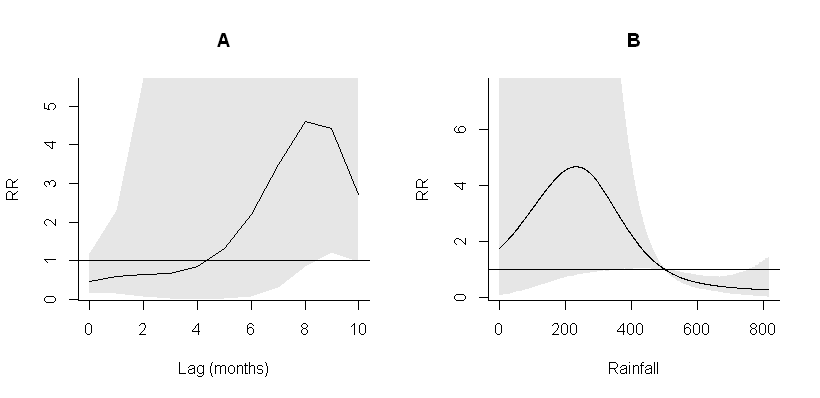


**Fig K**. Results of multivariable analysis of maximum RR of dengue incidence and climate for rainfall adjusting for mean temperature and relative humidity in Yangon Region. A. Lag response curve, association with rainfall at 250mm, centered at 500mm. B. Maximum associated risks at lag 8 months across different rainfall levels.


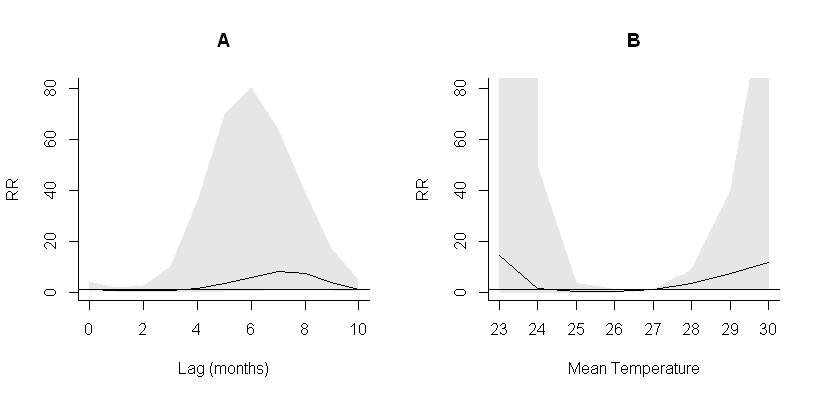


**Fig L**. Results of multivariable analysis of maximum RR of dengue incidence and climate for mean temperature adjusting for rainfall in Rakhine State. A. Lag response curve, association with mean temperature at 29°C, centered at 27°C. B. Maximum associated risks at lag 8 months across different temperatures.


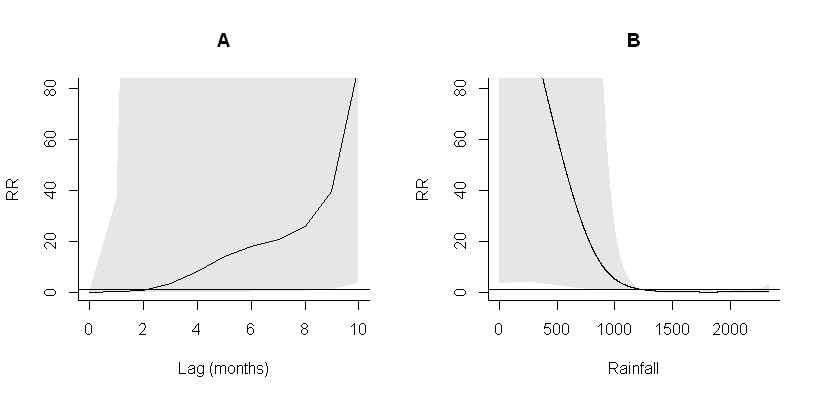


**Fig M**. Results of multivariable analysis of maximum RR of dengue incidence and climate for rainfall adjusting for mean temperature in Rakhine State. A. Lag response curve, association with rainfall at 350mm, centered at 1250mm. B. Maximum associated risks at lag 10 months across different rainfall levels.


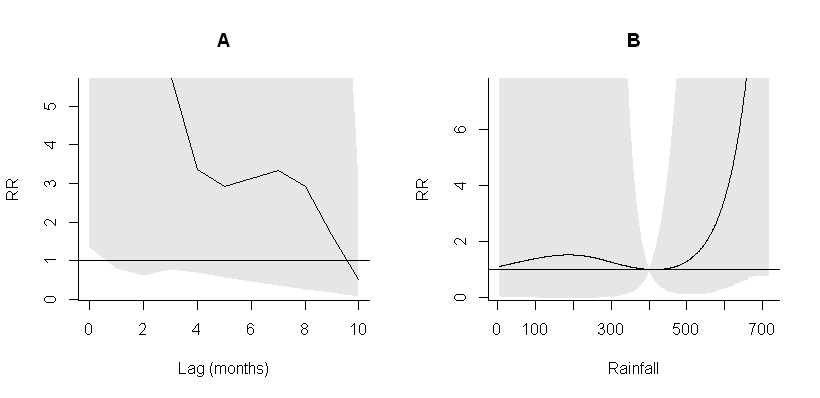


**Fig N**. Results of multivariable analysis of maximum RR of dengue incidence and climate for rainfall adjusting for relative humidity in Tanintharyi Region. A. Lag response curve, association with rainfall at 700mm, centered at 400mm. B. Maximum associated risks at lag 8 months across different rainfall levels.


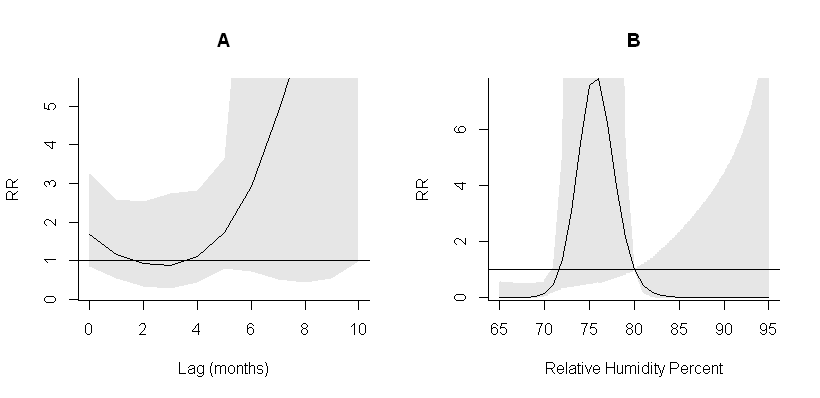


**Fig O**. Results of multivariable analysis of maximum RR of dengue incidence and climate for relative humidity adjusting for rainfall in Tanintharyi Region. A. Lag response curve, association with relative humidity at 76%, centered at 80%. B. Maximum associated risks at lag 9 months across different humidity levels.

**
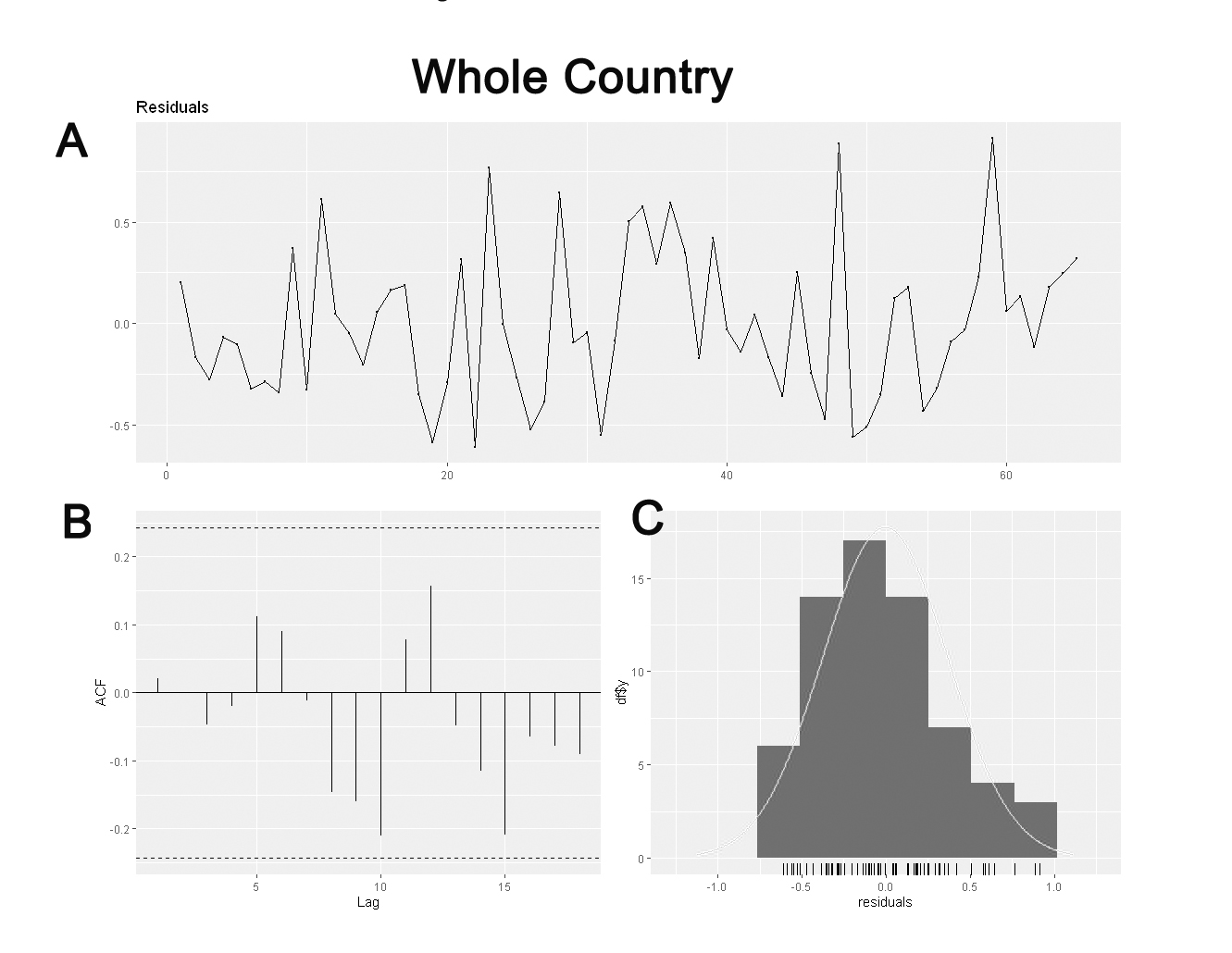
**

**Fig P.** Residuals of incidence prediction results using ARIMA model without weather variables for the whole country. Residuals of the model by month, B. Autocorrelation plot (ACF), C. Histograms of the residuals.


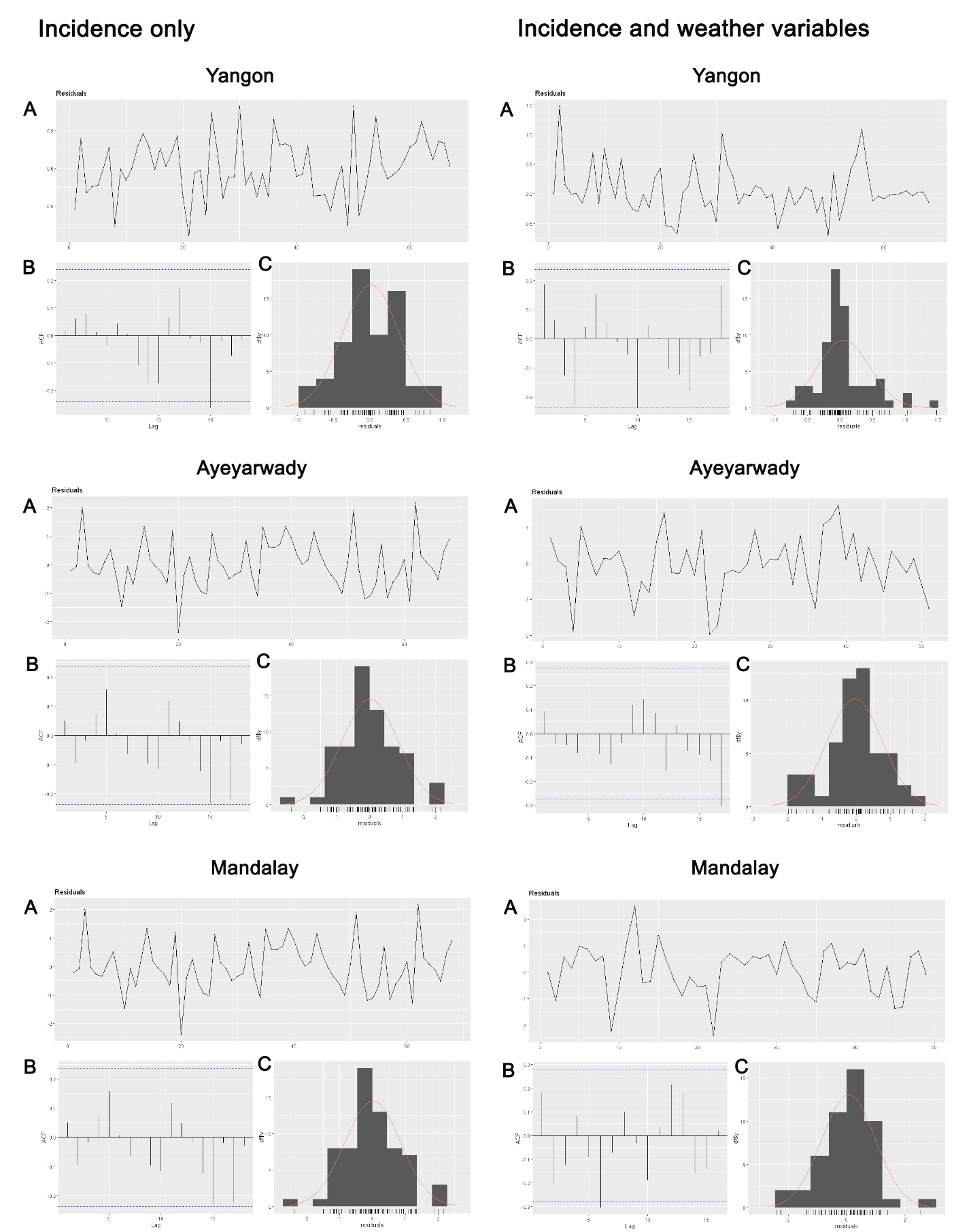


**Fig Q.** Residuals of incidence prediction results using ARIMA model without (left) and with (right) weather variables for Yangon, Mandalay, Ayeyarwady. Residuals of the model by month, B. Autocorrelation plot (ACF), C. Histograms of the residuals.

**Table A**. Annual dengue incidence per 100,000 population by State and Region.


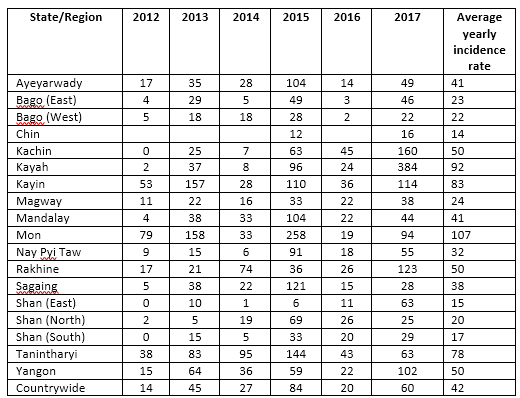


**Table B**. Annual dengue case fatality rates by State and Region.

**
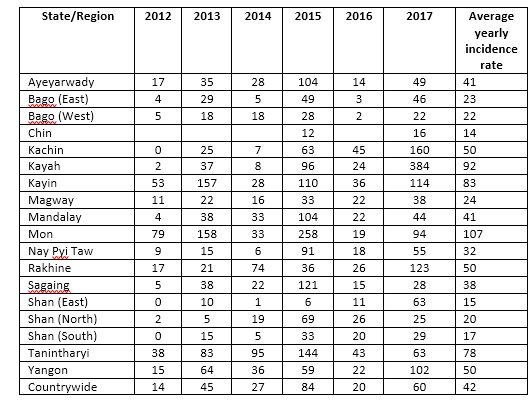
**

**Table C**. Reported dengue serotypes from 1999 to June 2018.


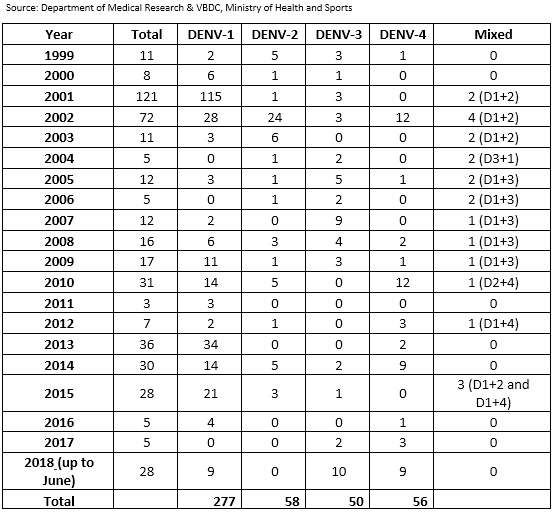


**Table D**. Correlation of dengue incidence per 100,000 population with different weather variables in Kachin, Kayah, Ayeyarwady, Mandalay, Sagaing, Tanintharyi and Yangon Regions.


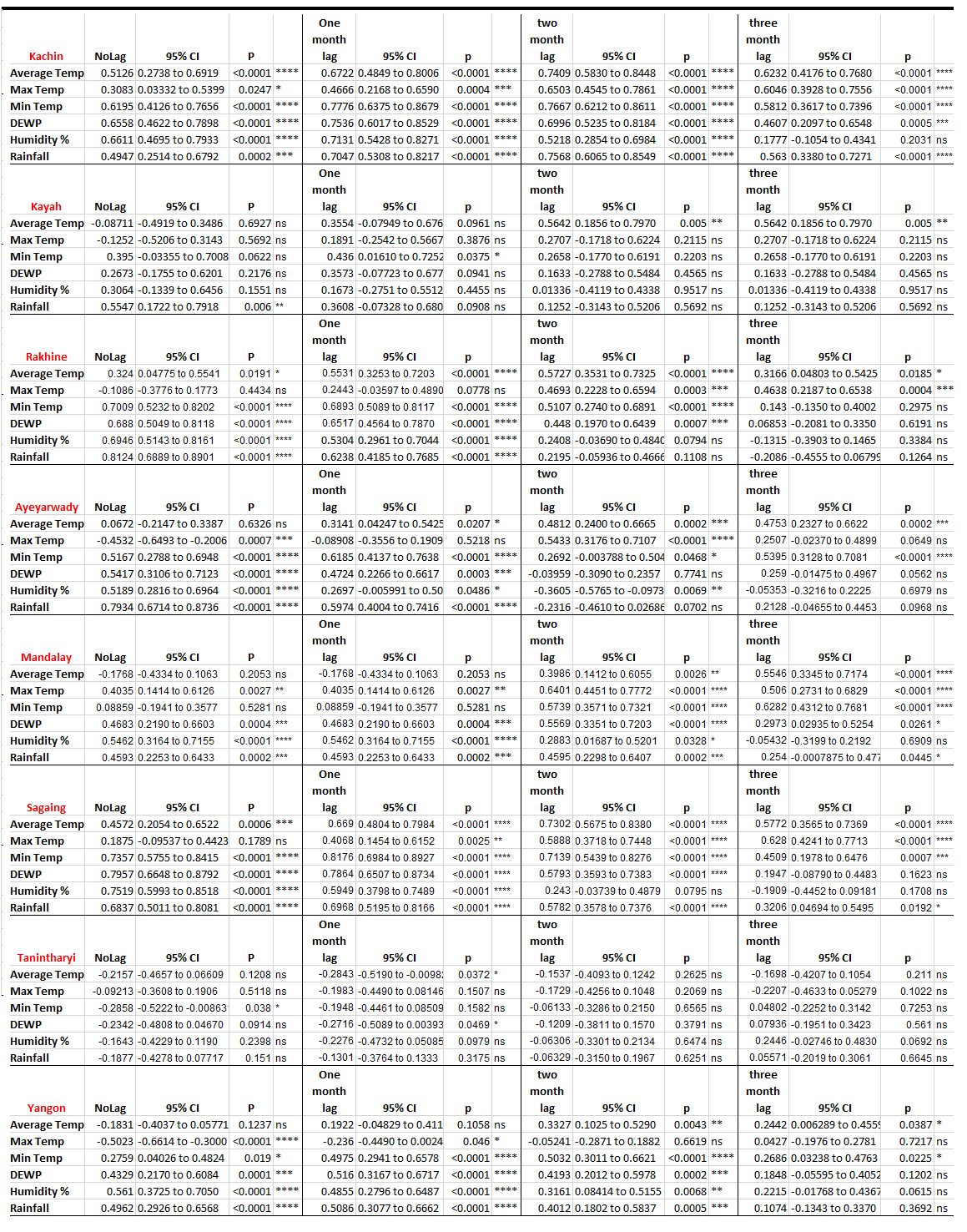


**Table E.** Correlation matrix of climate variables for Yangon Region

| Yangon | Rainfall | Mean Temp. | Max Temp. | Min Temp. | Dewpoint Temp. | Humidity |
| --- | --- | --- | --- | --- | --- | --- |
| Rainfall | 1 | -0.19 | -0.7 | 0.53 | 0.69 | 0.79 |
| Mean Temp. | -0.19 | 1 | 0.76 | 0.58 | 0.27 | -0.23 |
| Max. Temp. | -0.7 | 0.76 | 1 | -0.04 | -0.34 | -0.72 |
| Min. Temp. | 0.53 | 0.58 | -0.04 | 1 | 0.82 | 0.53 |
| Dewpoint Temp. | 0.69 | 0.27 | -0.34 | 0.82 | 1 | 0.87 |
| Humidity | 0.79 | -0.23 | -0.72 | 0.53 | 0.87 | 1 |

**Table F.** Correlation matrix of climate variables for Rakhine State

| Rakhine | Rainfall | Mean Temp. | Max Temp. | Min Temp. | Dewpoint Temp. | Humidity |
| --- | --- | --- | --- | --- | --- | --- |
| Rainfall | 1 | 0.21 | -0.24 | 0.54 | 0.47 | 0.53 |
| Mean Temp. | 0.21 | 1 | 0.81 | 0.87 | 0.79 | 0.41 |
| Max. Temp. | -0.24 | 0.81 | 1 | 0.45 | 0.47 | 0.07 |
| Min. Temp. | 0.54 | 0.87 | 0.45 | 1 | 0.85 | 0.59 |
| Dewpoint Temp. | 0.47 | 0.79 | 0.47 | 0.85 | 1 | 0.88 |
| Humidity. | 0.53 | 0.41 | 0.07 | 0.59 | 0.88 | 1 |

**Table G.** Correlation matrix of climate variables for Thanintharyi Region

| Thanintharyi | Rainfall | Mean Temp. | Max Temp. | Min Temp. | Dewpoint Temp. | Humidity |
| --- | --- | --- | --- | --- | --- | --- |
| Rainfall | 1 | -0.48 | -0.79 | 0.4 | 0.26 | 0.51 |
| Mean Temp. | -0.48 | 1 | 0.87 | 0.5 | 0.23 | -0.28 |
| Max. Temp. | -0.79 | 0.87 | 1 | 0.04 | 0.03 | -0.42 |
| Min. Temp. | 0.4 | 0.5 | 0.04 | 1 | 0.59 | 0.33 |
| Dewpoint Temp. | 0.26 | 0.23 | 0.03 | 0.59 | 1 | 0.87 |
| Humidity | 0.51 | -0.28 | -0.42 | 0.33 | 0.87 | 1 |

**Table H.** Correlation matrix of climate variables for Ayeyarwady Region

| Ayeyarwady | Rainfall | Mean Temp. | Max Temp. | Min Temp. | Dewpoint Temp. | Humidity |
| --- | --- | --- | --- | --- | --- | --- |
| Rainfall | 1 | -0.2 | -0.61 | 0.45 | 0.42 | 0.56 |
| Mean Temp. | -0.2 | 1 | 0.82 | 0.67 | 0.37 | -0.16 |
| Max Temp. | -0.61 | 0.82 | 1 | 0.16 | -0.03 | -0.48 |
| Min. Temp. | 0.45 | 0.67 | 0.16 | 1 | 0.79 | 0.47 |
| Dewpoint Temp. | 0.42 | 0.37 | -0.03 | 0.79 | 1 | 0.86 |
| Humidity | 0.56 | -0.16 | -0.48 | 0.47 | 0.86 | 1 |

**Table I.** Correlation matrix of climate variables for Sagaing Region

| Sagaing | Rainfall | Mean Temp. | Max Temp. | Min Temp. | Dewpoint Temp. | Humidity |
| --- | --- | --- | --- | --- | --- | --- |
| Rainfall | 1 | 0.63 | 0.34 | 0.78 | 0.87 | 0.72 |
| Mean Temp. | 0.63 | 1 | 0.9 | 0.92 | 0.79 | 0.25 |
| Max Temp. | 0.34 | 0.9 | 1 | 0.67 | 0.48 | -0.15 |
| Min. Temp. | 0.78 | 0.92 | 0.67 | 1 | 0.94 | 0.58 |
| Dewpoint Temp. | 0.87 | 0.79 | 0.48 | 0.94 | 1 | 0.79 |
| Humidity | 0.72 | 0.25 | -0.15 | 0.58 | 0.79 | 1 |

**Table J**. Comparisons of different regions’ ARIMA model order with and without covariates for the whole country, and for Yangon, Mandalay and Ayeyarwady Regions.

| No |  | **ARIMA order** | **MSE** | **R^2^** | **AIC** |
| --- | --- | --- | --- | --- | --- |
| 1.1 | Whole country | ARIMA(2,0,2) with non-zero mean | 0.129 | 0.887 | 70.38 |
| 1.2 | Whole country with seasonal component | ARIMA with only seasonal component | 0.196 | 0.871 | 105.9 |
|  |  |  |  |  |  |
| 2.1 | Yangon without weather covariate | ARIMA(0,0,3) with non-zero mean | 0.107 | 0.762 | 76.35 |
| 2.3 | Yangon with weather covariate | Regression with ARIMA(0,1,0) errors | 0.046 | 0.906 | 78.67 |
| 2.4 | Yangon with seasonal component | ARIMA with only seasonal component | 0.574 | 0.634 | 141.86 |
|  |  |  |  |  |  |
| 3.1 | Mandalay without weather covariate | ARIMA(2,0,1)with zero mean | 0.478 | 0.873 | 121.24 |
| 3.2 | Mandalay with weather covariate | Regression with ARIMA(1,0,0) errors | 0.462 | 0.890 | 132.41 |
| 3.3 | Mandalay seasonal component | ARIMA with only seasonal component | 1.756 | 0.606 | 155.6 |
|  |  |  |  |  |  |
| 4.1 | Ayeyarwaddy without weather covariate | ARIMA(2,0,1)with zero mean | 0.478 | 0.874 | 121.25 |
| 4.2 | Ayeyarwaddy with weather covariates | Regression with ARIMA(1,0,0)errors | 0.462 | 0.888 | 132.41 |
| 4.3 | Ayeyarwady with and seasonal component | ARIMA with only seasonal component | 1.846 | 0.648 | 98.99 |

**Table K**. Prediction comparisons of observed and predicted data with the best model and seasonality alone (first seasonal autoregressive and difference order) for the whole country and Mandalay Region, Mon State, Yangon Region and Ayeyarwady Region.


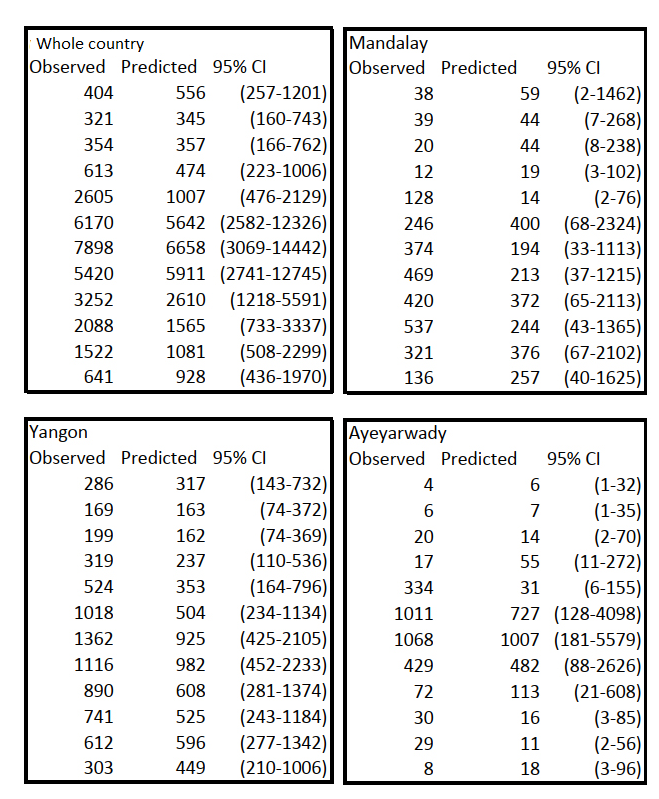

Supplement: S1 Text — Fig A in S1 Text. Proportion of dengue cases in urban and rural areas. Fig B in S1 Text. State/Region level monthly dengue cases and number of deaths. Fig C in S1 Text. Dengue severity grades by States and Regions in 2016 and 2017. Source and terms of use for shapefile: https://geonode.themimu.info/layers/geonode%3Ammr_polbnda_adm1_250k_mimu. Fig D in S1 Text. Overall association of univariate analysis with minimum temperature, mean temperature, maximum temperature, rainfall and relative humidity percent with dengue incidence in Yangon Region. Fig E in S1 Text. Lag response curves of overall relative risks of each meteorological variable with monthly dengue incidence per 100,000 population for Ayerarwady Region. Fig F in S1 Text. Lag response curves of overall relative risks of each meteorological variable with monthly dengue incidence per 100,000 population for Mandalay Region. Fig G in S1 Text. Lag response curves of overall relative risks of each meteorological variable with monthly dengue incidence per 100,000 population for Rakhine State. Fig H in S1 Text. Lag response curves of overall relative risks of each meteorological variable with monthly dengue incidence per 100,000 population for Sagaing Region. Fig I in S1 Text. Lag response curves of overall relative risks of each meteorological variable with monthly dengue incidence per 100,000 population for Thanintharyi Region. Fig J in S1 Text. Results of multivariable analysis of maximum RR of dengue incidence and climate for relative humidity percent adjusting for mean temperature and rainfall and in Yangon Region. A. Lag response curve, association with relative humidity at 76%, centered at 82.5%. B. Maximum associated risks at lag 6 months across different humidity levels. Fig K in S1 Text. Results of multivariable analysis of maximum RR of dengue incidence and climate for rainfall adjusting for mean temperature and relative humidity in Yangon Region. A. Lag response curve, association with rainfall at 250mm, [file pntd.0011331.s002.docx]
